# Efficient α-selective chlorination of phenylacetic acid and its *para*-substituted analogues†

**DOI:** 10.1039/d5ra00198f

**Published:** 2025-04-17

**Authors:** Camillo Morano, Alessandro Giraudo, Gabriella Roda, Edoardo Armano, Giulia Nasta, Massimiliano Sipala, Marco Pallavicini, Cristiano Bolchi

**Affiliations:** a Dipartimento di Scienze Farmaceutiche, Università Degli Studi di Milano Via Mangiagalli 25 Milano I-20133 Italy cristiano.bolchi@unimi.it

## Abstract

α-Chlorophenylacetic acids are synthons with great potential but are limited by the lack of a simple and generalizable method for their preparation from readily available precursors. Therefore, relying on the commercial availability of phenyl acetic acid and a series of *para*-substituted phenylacetic acids, we explored the practicability of their direct α-selective chlorination without the competing electrophilic aromatic chlorination. Indeed, treatment of these substrates with catalytic PCl_3_ and a slight excess of trichloroisocyanuric acid (TCCA) under solvent-free conditions rapidly provided the desired products in high yields with the only condition being that substituents that strongly activate electrophilic aromatic substitution were absent. An efficient preparative method for α-chlorinate phenylacetic acid and its analogues bearing electron-withdrawing or weakly electron-donating *para*-substituents, such as NO_2_, CN, CF_3_, COOMe, halogen, and alkyl, was thus developed, making these synthetic intermediates more accessible and exploitable.

## Abstract 2

α-Chlorophenylacetic acids are synthons with great potential but are limited by the lack of a simple and generalizable method for their preparation from readily available precursors.

## Graphical abstract

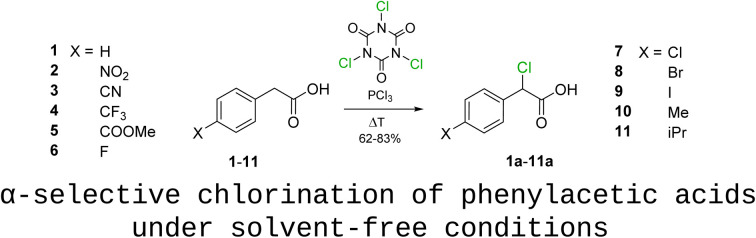

α-Chlorocarboxylic acids and their derivatives are valuable synthons. *Via* amidation, they afford α-chloroamides, which are versatile building blocks for the synthesis of a variety of molecules.^1^ Nucleophilic substitution of chlorine gives access to α-hydroxy,^2^ α-amino,^3^ α-mercapto,^4^ and other α-halo acids,^5^ and their reaction with carbonyl compounds leads to α,β-epoxy esters.^6^

Hell–Volhard–Zelinsky (HVZ) halogenation with molecular halogens and catalytic phosphorus trihalides represents the classical approach to synthesize these compounds. According to the generally accepted mechanism, it occurs through the initial formation of an aliquot of acyl halide, *via* phosphorus trihalide, from the starting carboxylic acid bearing at least one α-proton. The enol form of the acyl halide is then halogenated at the α-carbon to the α-halo acyl halide that, through reaction with excess unreacted carboxylic acid, gets converted into the desired α-halocarboxylic acid and regenerates the catalyzing acyl halide.^7^

To halogenate the enol form of the acyl halide, in place of molecular halogen, a halogenating agent can be used, which is able to furnish halogen atoms that are more positive and electrophilic than molecular halogen. With respect to such an ability for chlorination, *N*-chloroamides and *N*-chloroimides occupy a prominent role owing to their ease of handling and storage.^8^ Among *N*-chlorinated reagents, trichloroisocyanuric acid (TCCA) exhibits some superior features such as high atom efficiency; low cost; high stability; high solubility in many organic solvents, in contrast to the insolubility of the byproduct of chlorination, cyanuric acid (CA); and low toxicity (Fig. 1).^9^

**Fig. 1:**
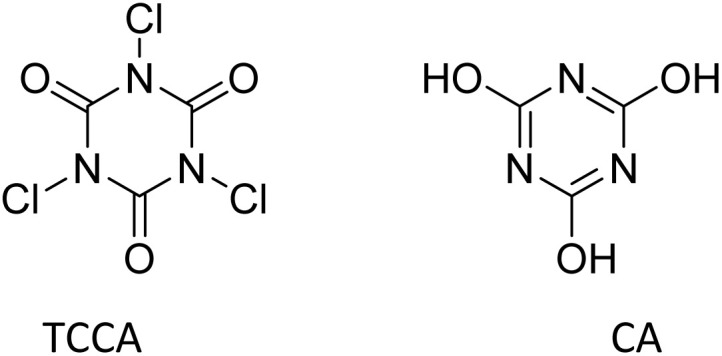
Structures of trichloroisocyanuric acid (TCCA) and cyanuric acid (CA).

TCCA has been used as an *N*-chlorinating reagent for amines and amides as well as a C-chlorinating reagent.^9,10^ For this purpose, it has been extensively studied for the chlorination of aromatic rings. If electron-rich, aromatic rings undergo monochlorination in acetonitrile by the action of TCCA, affording mixtures of regioisomers.^11^ In the presence of an acid catalyst, non-activated aromatic rings too are monochlorinated by TCCA under solvent-free and ball-milling conditions.^12^ C-Chlorination by TCCA can also occur at the benzylic position of alkyl aromatic hydrocarbons,^9^ the α-position of carbonyl^13^ and carboxyl compounds^14^ and the 2-position of 1,3-dicarbonyl compounds.^15^

Among α-chlorocarboxylic acids, α-chlorophenylacetic acids occupy a preeminent position for their potential as polyvalent synthons. However, the classical HVZ approach has never been applied to the preparation of α-chlorophenylacetic acid (1a) and its ring-substituted analogues. The reason lies in the HVZ mechanism necessitating electrophilic attack by a positive halogen atom, delivered by molecular halogen or an *N*-haloimidic halogenating agent, that can involve the enol form of the acyl halide but also, as extensively exemplified in the literature,^16^ the aromatic ring. The aforementioned chlorination ability of different nucleophilic carbons by TCCA provides a clear indication of how difficult α-selective chlorination of phenylacetic acids may be. Indeed, unlike straight-chain, saturated α-chloro carboxylic acids, α-chlorophenylacetic acids lack a simple and generalizable method of preparation from readily available precursors to date and relatively few examples of ring-substituted α-chlorophenylacetic acids have been reported in the literature.

Given these premises, the HVZ α-chlorination of phenylacetic acids seemed hardly practical. Nevertheless, we decided to explore the reactivity of phenylacetic acid (1) and a series of *para*-substituted phenylacetic acids (2–13), readily available from commercial sources, under HVZ chlorination conditions as opposed to excluding their α-selective chlorination potential *a priori* (Fig. 2, bottom). In fact, competition between the two possible electrophilic C-halogenations could be expected with the outcome being dependent on the presence of substituents capable of affecting the ring electron-richness through electron-withdrawing or electron-donating effect.

**Fig. 2:**
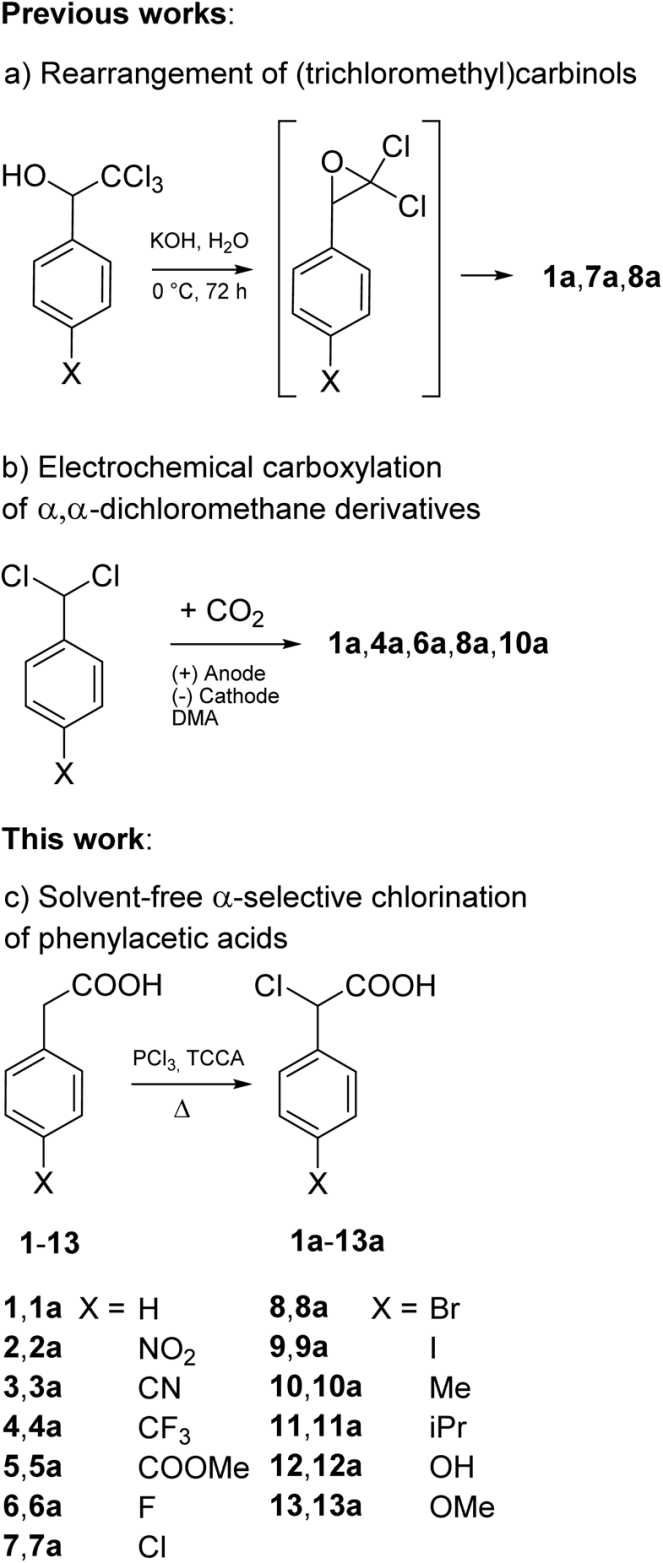
(a) and (b) Previously reported general methods for the synthesis of α-chlorophenylacetic acids; (c) phenylacetic acids subjected to chlorination with TCCA under solvent-free conditions in this work.

Our benchmark was 1 and our experiments of chlorination with TCCA started from this substrate. A survey of the literature revealed that the main methods of preparation of α-chlorophenylacetic acid (1a) are substitution of the hydroxyl group of methyl mandelate by chlorine followed by ester hydrolysis^17^ and the α-chlorination of phenylacetaldehyde with diphenic acid CuCl_2_ complex followed by oxidation with H_2_O_2_.^18^ Such methods exploit the availability of specific starting materials, and they are not generalizable or they haven't been generalized to the direct preparation of other ring substituted α-chlorophenylacetic acids.

Besides these two methods, two other procedures have been developed and extended for the preparation of other α-chlorinated phenylacetic acid analogues: the rearrangement of phenyl(trichloromethyl)carbinol by reaction with cold aqueous potassium hydroxide with 68% yield^19^ (Fig. 2a) and, very recently, the electrochemical carboxylation of α,α-dichlorophenylmethane with 44% yield (Fig. 2b).^20^ However, both the procedures require that the starting substrates are previously prepared: the former by Friedel–Crafts reaction of benzene or benzene derivatives with chloral,^18^ the latter by chlorination of aromatic aldehydes with PCl_5_.^19^ Furthermore, the rearrangement of trichloromethylcarbinols needs several days to afford α-chloroacetic acids in moderate yields while the electrochemical carboxylation features only modest yields, reported on one mmol scale.

Wanting to explore the output of a HVZ procedure applied to 1, we decided to use TCCA as the chlorinating agent under solvent-free conditions working at melting temperatures of the mixture. We performed the reactions on 1 g (7.34 mmol) of 1 using 0.1 mol of PCl_3_ and 0.5 mol of TCCA per mol of 1. Although TCCA can transfer all three chlorines to the substrate, based on previous reports on the chlorination of carbonyl and carboxyl compounds,^12,14^ we employed a slight excess of the chlorinating agent (0.5 mol instead of 0.33 mol TCCA/mol of 1). While the amounts of PCl_3_ and TCCA were maintained at these values across all the experiments, the mode of addition for both reagents, the reaction time and temperature were optimized. For the addition modes, three main protocols were adopted: (i) mixing 1, PCl_3_ and TCCA at room temperature and then heating to 85 °C (Table 1, protocol A), (ii) melting 1 by heating to 85 °C, addition of PCl_3_, cooling to room temperature, addition of TCCA and then heating to 85 °C again (Table 1, protocol B), (iii) mixing 1 and PCl_3_ at room temperature, heating the mixture to 85 °C and successive portion-wise addition of TCCA at the same temperature (Table 1, protocol C). In the second and third mode, 1 and PCl_3_ were allowed to react at 85 °C for 10 min and, after addition of TCCA, at room temperature or at 85 °C, the reaction at 85 °C was protracted over times varying from 1.5 to 6 h. The third procedure with a 1.5 h reaction at 85 °C after TCCA addition gave the best degree of conversion (∼100%) and was successively applied, on the same 1 g scale, to the other *para*-substituted phenylacetic acids adjusting the reaction temperature to melt the initial substrate/PCl_3_ mixture. Crude 1a resulted from filtering off cyanuric acid, precipitated by addition of ethyl acetate to the reaction mixture, and concentration of the filtrate previously washed with metabisulfite. It was purified by flash chromatography to give pure 1a as a white solid (1.04 g) with 83% yield.

**Table 1:** Optimization of the conversion of 1 into 1a through reaction with PCl_3_ (0.1 mol per mol of 1) and TCCA (0.5 mol per mol of 1) under solvent-free conditions

Protocol	Times, temperatures and addition modes	Conv (%)
A	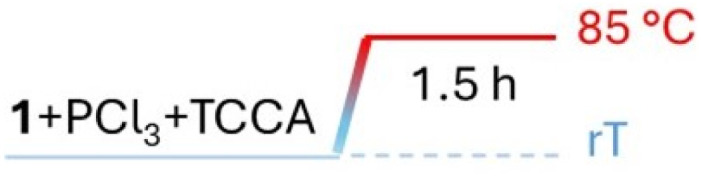	65
B	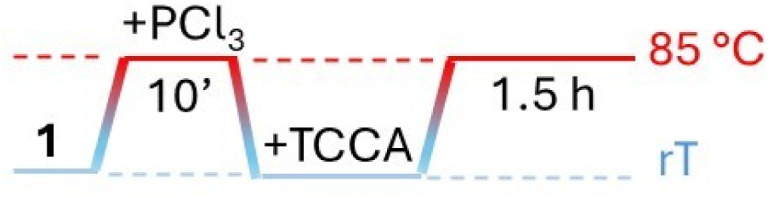	50
C	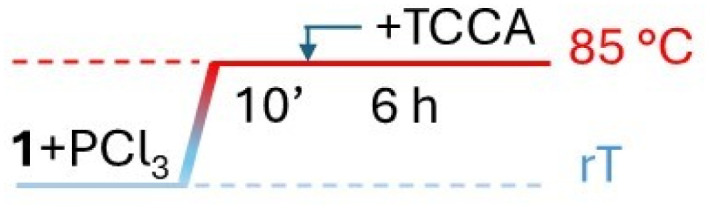	92
C	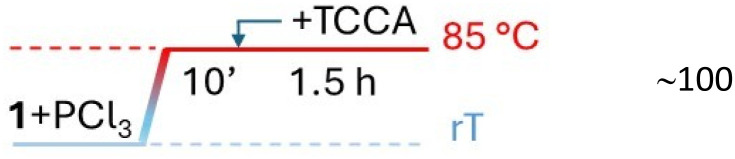	∼100

In all the reaction trials, the conversion degree of 1 into 1a could be easily determined by ^1^H NMR analysis as the crude products isolated as described were mixtures of only two main components, 1a and residual 1. The mole fractions of 1a and 1 could be reliably drawn from the integration of the widely separated singlets of CHCl and CH_2_ at 5.38 and 3.65 *δ* ppm, respectively. We did not observe aromatic chlorination, which, in the presence of a substituent that weakly activates electrophilic substitution such as CH_2_COOH,^21^ could not be excluded *a priori*. Aromatic chlorination became instead detectable when investigating the effect of lowering the amount of PCl_3_ from 0.1 to 0.075, 0.05 and 0.025 mol per mol of 1 in the third procedure protocol. The use of less PCl_3_ resulted in a progressively lower conversion of 1 (92, 86 and 46% respectively) and, most notably, a gradually decreasing yield of 1a and a corresponding gradually increasing yield *o*- and *p*-chlorophenylacetic acid, indicated in the ^1^H NMR spectra of the isolated crudes by the appearance of two additional singlets attributable to CH_2_ of *o*-chlorophenylacetic acid (at 3.82 *δ* ppm) and CH_2_ of *p*-chlorophenylacetic acid (at 3.62 *δ* ppm). Notably, an even lower conversion degree and more complex mixture of products, including among others 1a and *o*- and *p*-chlorophenylacetic acid, were obtained when the reaction was conducted in refluxing acetonitrile even when the amount of PCl_3_ was left unchanged. These results show that the solvent-free conditions and an adequate PCl_3_/substrate ratio are decisive for the competitiveness of the chlorination at the α position requiring the initial *in situ* conversion of 1 into phenylacetyl chloride. Consistently, we observed no α-chlorination in the absence of PCl_3_. Consequently, it can be assumed that the enol form of phenylacetyl chloride is then halogenated by TCCA to α-chlorophenylacetyl chloride, which would convert, according to HVZ mechanism, to 1a by reaction with excess unreacted 1 regenerating phenylacetyl chloride. As shown in Scheme 1, an α-chlorination cycle would thereby be established while 1 remains available. After 1 is consumed, residual α-chlorophenylacetyl chloride would be hydrolysed to 1a in the reaction work-up.

**Scheme 1:**
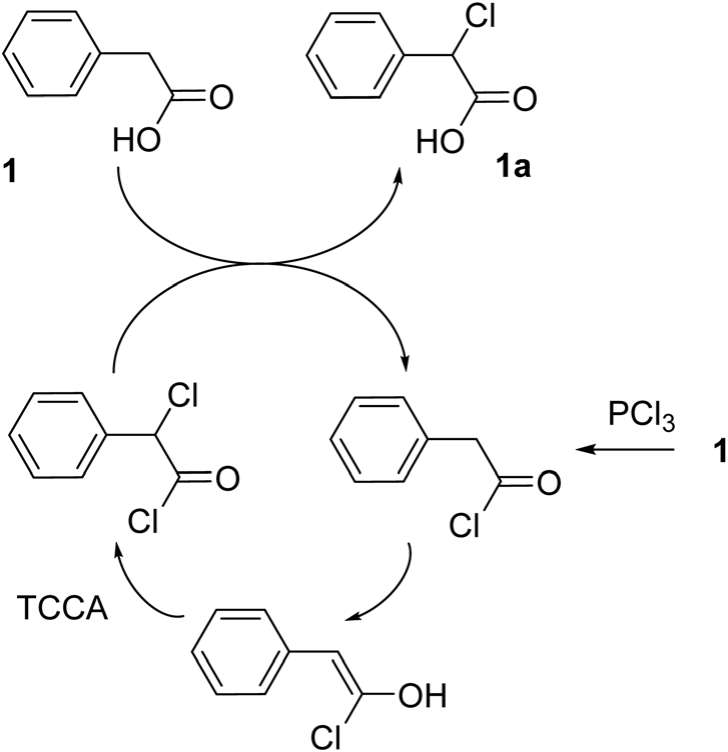
Proposed reaction mechanism of α-chlorination of phenylacetic acid with TCCA in the presence of PCl_3_ under solvent-free conditions.

The high yield, ease and speed of the α-chlorination of 1 with TCCA under solvent-free conditions, together with the good availability of ring-substituted phenylacetic acids, prompted us to verify the generalizability of this procedure to the α-chlorination of *para*-substituted phenylacetic acids. Among the ring-substituted phenylacetic acids, *para*-substituted substrates are the most widely available. Furthermore, the screening of such substrates would allow for an increased appreciation of the importance, for the α-regioselective chlorination, of the effect that the ring substituent exerts, with perfect symmetry to the reaction center, on the electron-richness of the ring and, in case of mesomeric effect, of the ring carbon adjacent to CH_2_ with exclusion of intramolecular steric and electrostatic interactions that could interfere with the reaction.

Our chlorination experiments on 1 showed that α-chlorination selectively occurs at the benzylic carbon with TCCA under solvent-free conditions without requiring the presence of substituents decreasing the electrophilicity of the aryl system nor the action of an acid catalyst, presumably due to the easy enolization of phenylacetyl chloride. On the basis of this evidence, it seemed appropriate to continue our investigation selecting a number of phenylacetic acids bearing *para*-substituents strongly deactivating electrophilic aromatic substitution, such as NO_2_ (2), CN (3) and CF_3_ (4), moderately deactivating, such as COOMe (5), weakly deactivating, such as F (6), Cl (7), Br (8) and I (9), and also weakly activating, such as methyl (10) and isopropyl (11). The results obtained with 1 promised similarly efficient α-chlorination of these substrates, with some uncertainty as to 10 and 11, due to the weak activation of electrophilic aromatic substitution and the presence of an additional benzylic position. 4-Hydroxy- and 4-methoxy-phenylacetic acid (12 and 13, respectively) were also included into the experiments in order to verify that *para*-substituents strongly activating electrophilic aromatic substitution and electron enriching the ring carbon adjacent to CH_2_ effectively hinder α-chlorination to 12a and 13a.

To our knowledge, preparations, with reported isolated yield, of the α-chlorophenylacetic acids 12a and 13a bearing *para*-substituents that strongly activate electrophilic aromatic substitution, OH and OMe respectively, are not described in the literature and neither are those of the α-chlorophenylacetic acids 2a, 3a, 5a and 9a, which instead bear *para*-substituents that deactivate electrophilic aromatic substitution (NO_2_, CN, COOMe and I, respectively). Of the remaining six, of which none bear strongly activating *para*-substituents, 7a and 8a have been obtained, with 44% and 49% yield respectively, by rearrangement of *p*-chloro- and *p*-bromo-phenyl(trichloromethyl)carbinol by reaction with cold aqueous potassium hydroxide during three days (Fig. 2a),^19^ whereas 4a, 6a, and 10a, with isolated yields ranging between a minimum 37% (4a) and a maximum 54% (10a), by electrochemical carboxylation of the corresponding *p*-trifluoromethyl, *p*-fluoro and *p*-methyl substituted α,α-dichlorotoluenes, in turn prepared from the corresponding p-substituted benzaldehydes (Fig. 2b).^20^ The method of electrochemical carboxylation has been applied also to obtain 8a achieving a yield (48%) analogous to that reported above (49%), resulting from the trichloromethylcarbinol rearrangement. No preparations are reported for 11a.

Wanting to extend the procedure developed for the α-chlorination of 1 to 2–11, we decided not to modify the quantities of the reactants nor the conditions. Nevertheless, we had to consider that, apart from 4, 6, 10, and 11, the other *p*-substituted phenylacetic acids melt at sensibly higher temperature than 1. Therefore, for the phenylacetic acids bearing NO_2_, CN, COOMe, Cl, Br, and I as a *para*-substituent, the temperature of the heating block was set higher than 85 °C (see Table 2) after adding PCl_3_ at room temperature to obtain a fluid and stirrable mass, to which TCCA could be added. Furthermore, in the cases of 10 and 11, PCl_3_ had to be increased to 0.2 mol and TCCA to 0.6 mol per mol of substrate, respectively, to achieve complete and high conversion.

**Table 2:** α-Chlorination of phenylacetic acids by TCCA under solvent-free conditions^a^

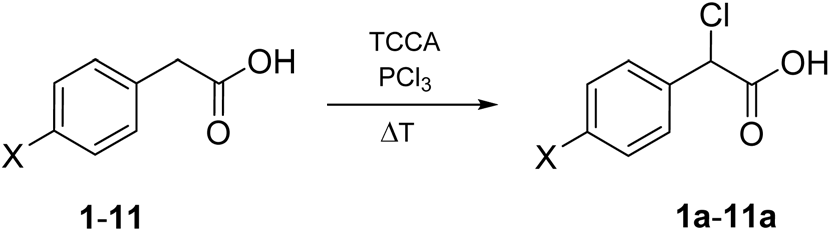				
X	Substrate	Product	*T* [°C]^b^	Yield [%]^c^
H	1	1a	85	83
NO_2_	2	2a	105	79
CN	3	3a	150	—d
CF_3_	4	4a	85	78
COOMe	5	5a	95	73
F	6	6a	85	78
Cl	7	7a	90	72
Br	8	8a	90	73
I	9	9a	110	67
Me	10	10a	85	62
^i^Pr	11	11a	85	71

a General reaction conditions: substrate (1 g) and PCl_3_ (0.1 or 0.2 mol PCl_3_/mol of phenylacetic acid) mixed at room temperature and allowed to react at the reported temperature for 10 min; TCCA (0.5 or 0.6 mol TCCA/mol of the substrate) added to the mixture at the reported temperature and allowed to react at the same temperature for 1.5 h.

b Reaction temperature.

c Isolated yield.

d Isolated as an unpurifiable crude (1.14 g from 1 g of 3) containing 3a as the major component.

To isolate crude 2a–11a, we replicated the very simple work-up adopted for 1a consisting of adding ethyl acetate, precipitating and filtering off cyanuric acid, washing with metabisulfite and concentrating the filtrate. As for 1a, the ^1^H NMR spectra showed that all the crudes were the desired α-chlorinated products containing traces or low amounts (≤0.08 mole fraction) of the starting acid, except for 10a, containing a ∼0.25 mole fraction of the byproduct resultant from the chlorination of both CH_2_ and CH_3_, and 11a, containing a ∼0.2 mole fraction of residual 11.

Purification of the crudes was accomplished by crystallization (2a, 5a, 7a, 8a, 9a), chromatography (4a and 6a) or vacuum distillation (10a and 11a). For 3a, analogously to what has been reported by Oudeyer,^20^ any attempt of purifying the crude, isolated, with a near quantitative yield, as a waxy solid containing a 0.08 mole fraction of residual 3, was unsuccessful either by crystallization or chromatography or vacuum distillation due to instability of the product under the purification conditions.

As expected, the phenylacetic acids 12 and 13 with *para*-substituents strongly activating electrophilic aromatic substitution could not provide the corresponding α-chloro acids. In our attempts, we observed that the reactions were vigorous with liberation of gas and formation of complex mixtures of products.

In conclusion, we have developed an efficient and easy method for α-selective HVZ chlorination of phenylacetic acid demonstrating that it can be successfully applied also to phenylacetic acids bearing electron-withdrawing or weakly electron donating substituents at the *para* position. From these findings, its extendibility to *meta*- and *ortho*-substituted analogues is foreseeable and experiments have been planned in that direction. The procedure has manifold advantageous features: versatility, solvent-free conditions, use of a sustainable chlorinating reagent such as TCCA, availability of the starting substrates, and ability to directly access a series of valuable synthons. To date, and to our knowledge, there are no reports on preparative methods to obtain α-chlorinated phenylacetic acids that are as simple and widely applicable.

## Data availability

Experimental data are available in the Electronic supplementary information (ESI): complete experimental procedures and characterization of new products, NMR spectra, and HRMS spectra for the new products (2a, 5a, 7a, 9a, and 11a).†

## Conflicts of interest

There are no conflicts to declare.

## Supplementary Material

RA-015-D5RA00198F-s001

RA-015-D5RA00198F-s002
