# Supplementary material for: Efficient α-selective chlorination of phenylacetic acid and its para-substituted analogues
Source: RSC Adv. 2025 Apr 17;15(16):12298–303. doi: 10.1039/d5ra00198f (PMC12004119; doi:10.1039/d5ra00198f)
Supplement: RA-015-D5RA00198F-s001 [file RA-015-D5RA00198F-s001.pdf]

Supplementary Information (SI) for RSC Advances.
This journal is © The Royal Society of Chemistry 2025

Supporting Information

# Efficient α-Selective Chlorination of Phenylacetic Acid and its *para*-Substituted Analogues

Camillo Morano,[a] Alessandro Giraudo,[a] Gabriella Roda,[a] Edoardo Armano,[a] Giulia Nasta,[a] Massimiliano Sipala,[a] Marco Pallavicini,[a] Cristiano Bolchi *[a]

Dipartimento di Scienze Farmaceutiche, Università degli Studi di Milano, via Mangiagalli 25, I-20133, Milano, Italy

## Table of Contents

1. General experimental details........................................2
2. General procedure for the preparation of **1a**, **2a**, **4a**, **5a**, **6a**, **7a**, **8a**, and **9a**..................2
3. General procedure for the preparation of **10a** and **11a**..........................4
4. References........................................5
5. 1H NMR, 13C NMR and HRMS spectra........................................6

## 1. General experimental details

Phenylacetic acids **1-11** were purchased from commercial sources. An aluminum reaction block was used as the heating source for reactions that required heating. Flash chromatography purifications were performed by using Sfär Silica D 60 μm cartridges. $^1$H NMR and $^{13}$C{1H}-NMR spectra were recorded in $CDCl_3$ at 300 and 75 MHz respectively, with a Varian Mercury 300 Spectrometer. Chemical shifts are reported in ppm relative to residual solvent as an internal standard. Melting points were determined by a Buchi Melting Point B-540 apparatus. Thin-layer chromatography (TLC) analyses were carried out on alumina sheets precoated with silica gel 60 F254. High Resolution Mass Spectra (HRMS) were acquired by direct infusion on a Waters Q-ToF Synapt G2-Si HDMS (Waters Corporation, Milford, MA, United States) operated in negative ElectroSpray Ionization (ESI-).

## 2. General procedure for the preparation of 1a, 2a, 4a, 5a,6a, 7a, 8a, and 9a

$PCl_3$ (0.3 equiv) was added to the appropriate phenylacetic acid (1 g) and the mixture was heated to the melting temperature (as below specified for each compound) in an aluminium reaction block and allowed to react for 10 min under stirring. At the same temperature, TCCA (1.5 equiv) was added portion wise over 15 min and allowed to react for 1.5 hours. The reaction mixture was cooled to room temperature, diluted with ethyl acetate and filtered. The filtrate was washed with 0.5 M aqueous solution of sodium metabisulfite and brine, dried over anhydrous sodium sulphate and concentrated under vacuum to give the crude product that was purified by crystallization of chromatography on silica gel.

*2-Chloro-2-phenylacetic acid (**1a**).*[1] Obtained from phenylacetic acid (**1**) (1 g, 7.34 mmol), $PCl_3$ (64 μL, 0.73 mmol) and TCCA (850 mg, 3.7 mmol) at 85 °C according to the general procedure. The crude product, a wax-like whitish solid (1.25 g), was purified by flash chromatography on silica gel eluting with dichloromethane/methanol/formic acid (95:5:1) to yield **1a** as a white solid (1.04 g, 83%): $R_f$ (dichloromethane/methanol 8:2) 0.51; mp 76.5-77.2 °C; $^1$H NMR (300 MHz, $CDCl_3$) $\delta$ 8.72 (br s, 1H), 7.54-7.50 (m, 2H), 7.41-7.38 (m, 3H), 5.38 (s, 1H); $^{13}$C NMR (75 MHz, $CDCl_3$) $\delta$ 174.3, 135.1, 129.7, 129.1, 128.1, 58.8.

*2-Chloro-2-(4-nitrophenyl)acetic acid (**2a**).* Obtained from 4-nitrophenylacetic acid (**2**) (1 g, 5.52 mmol), $PCl_3$ (48 μL, 0.55 mmol) and TCCA (641 mg, 2.76 mmol) at 105 °C according to the general procedure. The crude product, a yellow solid (1.16 g), was purified by crystallization from cyclohexane/ethyl acetate (9:1 v/v) to yield **2a** as a light yellow solid (0.94 g, 79%): $R_f$ (dichloromethane/methanol 8:2) 0.55; mp 132.1-133.3 °C; $^1$H NMR (300 MHz, $CDCl_3$) $\delta$ 8.26 (d, $J$=8.5 Hz, 2H), 7.71 (d, $J$=8.5 Hz, 2H), 6.99 (br s, 1H), 5.45 (s, 1 H); $^{13}$C NMR (75 MHz, $CDCl_3$) $\delta$ 171.8, 148.7, 141.7, 129.3, 124.3, 57.4; HRMS (ESI$^-$) $m/z$ calcd for $C_7H_5ClNO_2$ $[M-COOH]^-$ 170.0008, found 170.0010.

*2-Chloro-2-(4-(trifluoromethyl)phenyl)acetic acid (**4a**).*[1] Obtained from 4-(trifluoromethyl)phenylacetic acid (**4**) (1 g, 4.90 mmol), $PCl_3$ (43 µL, 0.49 mmol) and TCCA (569 mg, 2.45 mmol) at 85 °C according to the general procedure. The crude product, a wax-like whitish solid (1.12 g), was purified by flash chromatography on silica gel (gradient of dichloromethane/methanol from 0% to 10% methanol) to yield **4a** as a white solid (910 mg, 78%): $R_f$ (dichloromethane/methanol 8:2) 0.57; mp 85.3-86.0 °C; $^1$H NMR (300 MHz, $CDCl_3$) $\delta$ 10.77 (br s, 1H), 7.66 (s, 4H), 5.42 (s, 1H); $^{13}$C NMR (75 MHz, $CDCl_3$) $\delta$ 173.3, 138.8, 131.9 (q, *J*=32.8 Hz), 128.6, 126.1 (q, *J*=3.7 Hz), 123.8 (q, *J*=272.4 Hz), 57.9.

*2-Chloro-2-(4-(methoxycarbonyl)phenyl)acetic acid (**5a**).* Obtained from 4-(methoxycarbonyl)phenylacetic acid (**5**) (1 g, 5.18 mmol), $PCl_3$ (45 µL, 0.52 mmol) and TCCA (602 mg, 2.59 mmol) at 95 °C according to the general procedure. The crude product, a wax-like white solid (1.11 g), was purified by trituration with cyclohexane to yield **5a** as a white solid (860 mg, 73%): $R_f$ (dichloromethane/methanol 8:2) 0.3; mp 77.7-78.9 °C; $^1$H NMR (300 MHz, $CDCl_3$) $\delta$ 9.46 (br s, 1H), 8.05 (d, *J*=8.0 Hz, 2H), 7.59 (d, *J*=8.0 Hz, 2H), 5.41 (s, 1H), 3.93 (s, 3 H); $^{13}$C NMR (75 MHz, $CDCl_3$) $\delta$ 172.4, 166.6, 139.9, 131.3, 130.3, 128.2, 58.2, 52.6; HRMS (ESI$^-$) *m/z* calcd for $C_9H_8ClO_2$ [M-COOH]$^-$ 183.0213, found 183.0218.

*2-Chloro-2-(4-fluorophenyl)acetic acid (**6a**).*[1] Obtained from 4-fluorophenylacetic acid (**6**) (1 g, 6.49 mmol), $PCl_3$ (57 µL, 0.65 mmol) and TCCA (754 mg, 3.25 mmol) at 85 °C according to the general procedure. The crude product, a wax-like yellow solid (1.16 g), was purified by flash chromatography on silica gel (gradient of dichloromethane/methanol from 0% to 10% methanol) to yield **6a** as a whitish solid (960 mg, 78%): $R_f$ (dichloromethane/methanol 8:2) 0.77; mp 82.4-83.3 °C; $^1$H NMR (300 MHz, $CDCl_3$) $\delta$ 10.40 (br s, 1H), 7.54-7.47 (m, 2H), 7.12-7.04 (m, 2 H), 5.36 (s, 1 H); $^{13}$C NMR (75 MHz, $CDCl_3$) $\delta$ 174.1, 163.5 (d, *J*=250 Hz), 130.9 (d, *J*=3.5 Hz), 130.2 (d, *J*=8.6 Hz), 116.2 (d, *J*=22.2 Hz), 58.0.

*2-Chloro-2-(4-chlorophenyl)acetic acid (**7a**).*[2] Obtained from 4-chlorophenylacetic acid (**7**) (1 g, 5.86 mmol), $PCl_3$ (51 µL, 0.59 mmol) and TCCA (681 mg, 2.93 mmol) at 90 °C according to the general procedure. The crude product, a colorless oil (1.13 g), was purified by crystallization from hexane to yield **7a** as a white solid (860 mg, 72%): $R_f$ (dichloromethane/methanol 8:2) 0.85; mp 76.9-77.7 °C; $^1$H NMR (300 MHz, $CDCl_3$) $\delta$ 10.32 (br s, 1H), 7.45 (d, *J*=8.6 Hz, 2H), 7.37 (d, *J*=8.6 Hz, 2H), 5.34 (s, 1H); $^{13}$C NMR (75 MHz, $CDCl_3$) $\delta$ 173.7, 135.9, 133.6, 129.5, 129.3, 58.0. HRMS (ESI$^-$) *m/z* calcd for $C_8H_5Cl_2O_2$ [M-H]$^-$ 202.9667, found 202.9669.

*2-Chloro-2-(4-bromophenyl)acetic acid (**8a**).*[2] Obtained from 4-bromophenylacetic acid (**8**) (1 g, 4.65 mmol), $PCl_3$ (41 µL, 0.47 mmol) and TCCA (542 mg, 2.33 mmol) at 90 °C according to the general procedure. The crude product, a wax-like whitish solid (1.26 g), was purified by crystallization from hexane to yield **8a** as a white solid (850 mg, 73%): $R_f$ (dichloromethane/methanol 8:2) 0.67; mp 77.4-

78.2 °C; $^1$H NMR (300 MHz, $CDCl_3$) $\delta$ 7.53 (d, $J$=8.5 Hz, 2H), 7.39 (d, $J$=8.5 Hz, 2H), 5.32 (s, 1H); $^{13}$C NMR (75 MHz, $CDCl_3$) $\delta$ 174.0, 134.0, 132.3, 129.8, 124.1, 58.0.

*2-Chloro-2-(4-iodophenyl)acetic acid (**9a**)*. Obtained from 4-iodophenylacetic acid (**9**) (1 g, 3.82 mmol), $PCl_3$ (33 µL, 0.38 mmol) and TCCA (443 mg, 1.91 mmol) at 110 °C according to the general procedure. The crude product, a yellow solid (1.04 g), was purified by crystallization from cyclohexane/ethyl acetate (9:1, v/v) to yield **9a** as a yellow solid (760 mg, 67%): $R_f$ (dichloromethane/methanol 8:2) 0.55; mp 108.8 °C (dec); $^1$H NMR (300 MHz, $CDCl_3$) $\delta$ 7.74 (d, $J$=8.4 Hz, 2H), 7.25 (d, $J$=8.4 Hz, 2H), 5.87 (br s, 1H), 5.30 (s, 1H); $^{13}$C NMR (75 MHz, $CDCl_3$) $\delta$ 172.6, 138.3, 134.9, 129.9, 95.9, 58.2, 52.6; HRMS (ESI$^-$) $m/z$ calcd for $C_8H_5ClIO_2$ [M-H]$^-$ 294.9023, found 294.9024.

## 3. General procedure for the preparation of 10a and 11a

The procedure was identical to the general procedure adopted for **1a**, **2a**, and **4a**-**9a**, but using 0.6 equivalents of $PCl_3$ instead of 0.3 and 1.8 equivalents of TCCA instead of 1.5 and purifying the crude product by vacuum distillation.

*2-Chloro-2-(p-tolyl)acetic acid (**10a**)*.[1] Obtained from *p*-tolylacetic acid (**10**) (1 g, 6.66 mmol), $PCl_3$ (116 µL, 1.33 mmol) and TCCA (934 mg, 4.02 mmol) at 85 °C according to the general procedure. The crude product, a wax-like white solid solid (1.16 g), was purified by distillation under vacuum (0.5 with a kugelrohr apparatus at 150-155 °C (0.5 mbar) to yield **10a** as a white solid (760 mg, 62%): $R_f$ (dichloromethane/methanol 8:2) 0.56; mp 81.5-82.3 °C; $^1$H NMR (300 MHz, $CDCl_3$) $\delta$ 7.40 (d, $J$=8.0 Hz, 2H), 7.20 (d, $J$=8.0 Hz, 2H), 5.36 (s, 1H), 2.36 (s, 3 H); $^{13}$C NMR (75 MHz, $CDCl_3$) $\delta$ 174.0, 139.9, 132.2, 129.8, 128.0, 58.7, 21.4.

*2-Chloro-2-(4-isopropylphenyl)acetic acid (**11a**)*. Obtained from 4-isopropylphenylacetic acid (**11**) (1 g, 5.61 mmol), $PCl_3$ (98 µL, 1.12 mmol) and TCCA (782 mg, 3.37 mmol) at 85 °C according to the general procedure. The crude product, a wax-like white solid solid (1.29 g), was purified by distillation under vacuum (0.5 mbar) with a kugelrohr apparatus at 150-155 °C to yield **11a** as a white solid (850 mg, 71%): $R_f$ (dichloromethane/methanol 8:2) 0.56; mp 76.4-77.6 °C; $^1$H NMR (300 MHz, $CDCl_3$) $\delta$ 7.43 (d, $J$=8.0 Hz, 2H), 7.25 (d, $J$=8.0 Hz, 2H), 5.37 (s, 1H), 2.92 (hep, $J$=6.9 Hz, 1H), 1.25 (d, $J$=6.9 Hz, 6H); $^{13}$C NMR (75 MHz, $CDCl_3$) $\delta$ 174.2, 151.1, 132.9, 128.6, 127.7, 59.2, 34.5, 24.4; HRMS (ESI$^-$) $m/z$ calcd for $C_{11}H_{12}ClO_2$ [M-H]$^-$ 211.0526, found 211.0530.

4. **References**


1. C. Maret, N. David, D. Pierrot, E. Leonel, V. Levacher, J. F. Briere, S. Oudeyer, Synthesis of α-chloroarylacetic acid via electrochemical carboxylation of α,α-dichloroarylmethane derivatives, *Molecules*, 2023, **28**, 6704.
2. W. Reeve, J. R. McKee, R. Brown, S. Lakshmanan, G. A. McKee, Studies on the rearrangement of (trichloromethyl)carbinols to α-chloroacetic acids, *Can. J. Chem.*, 1980, **58**, 485-493.

5. **1H NMR, 13C NMR and HRMS spectra**

***2-Chloro-2-phenylacetic acid (1a)***

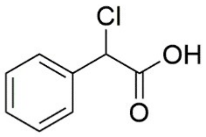


crude

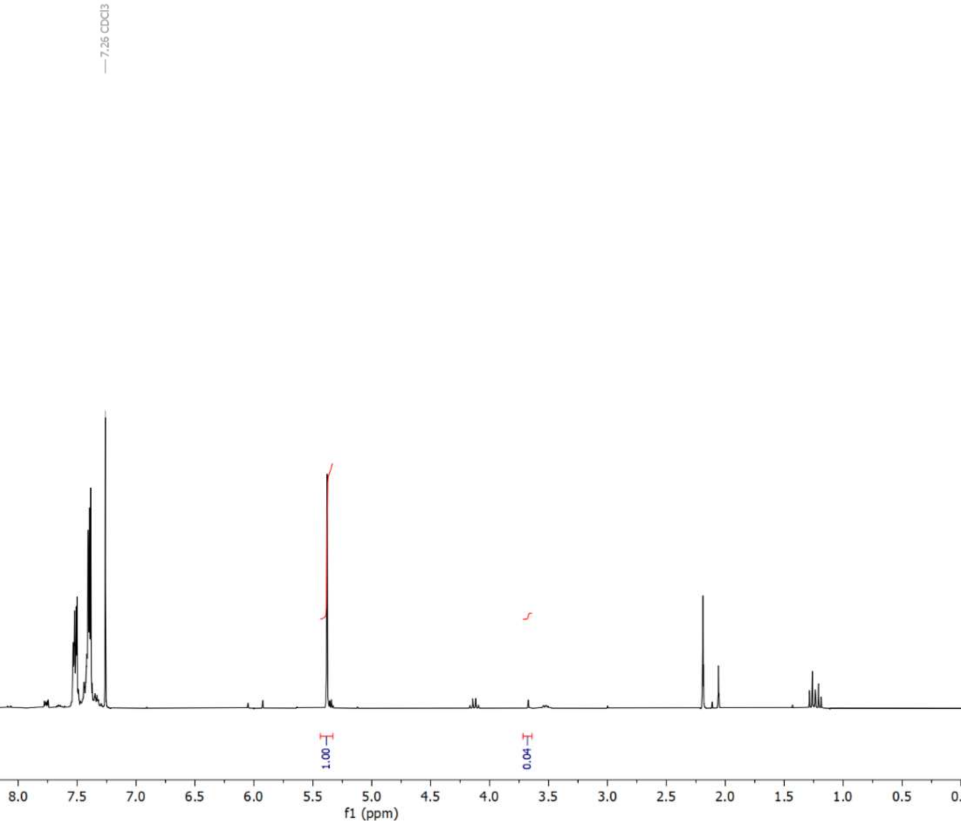


purified

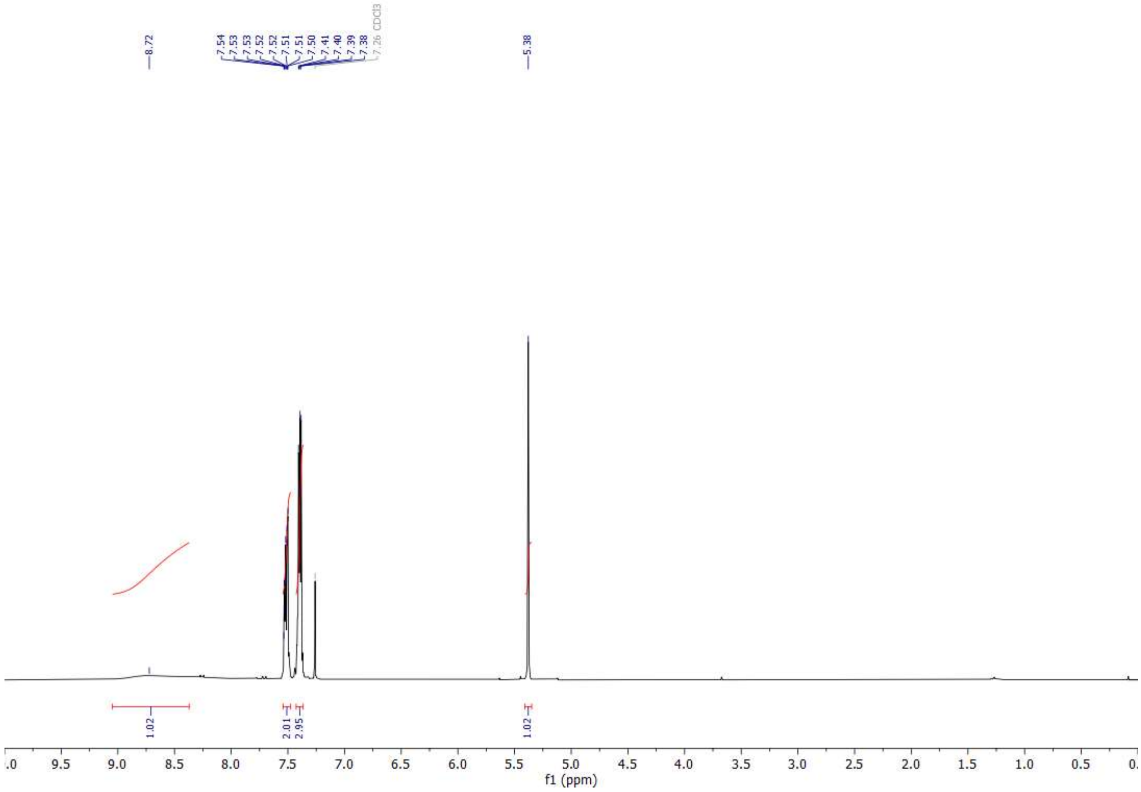

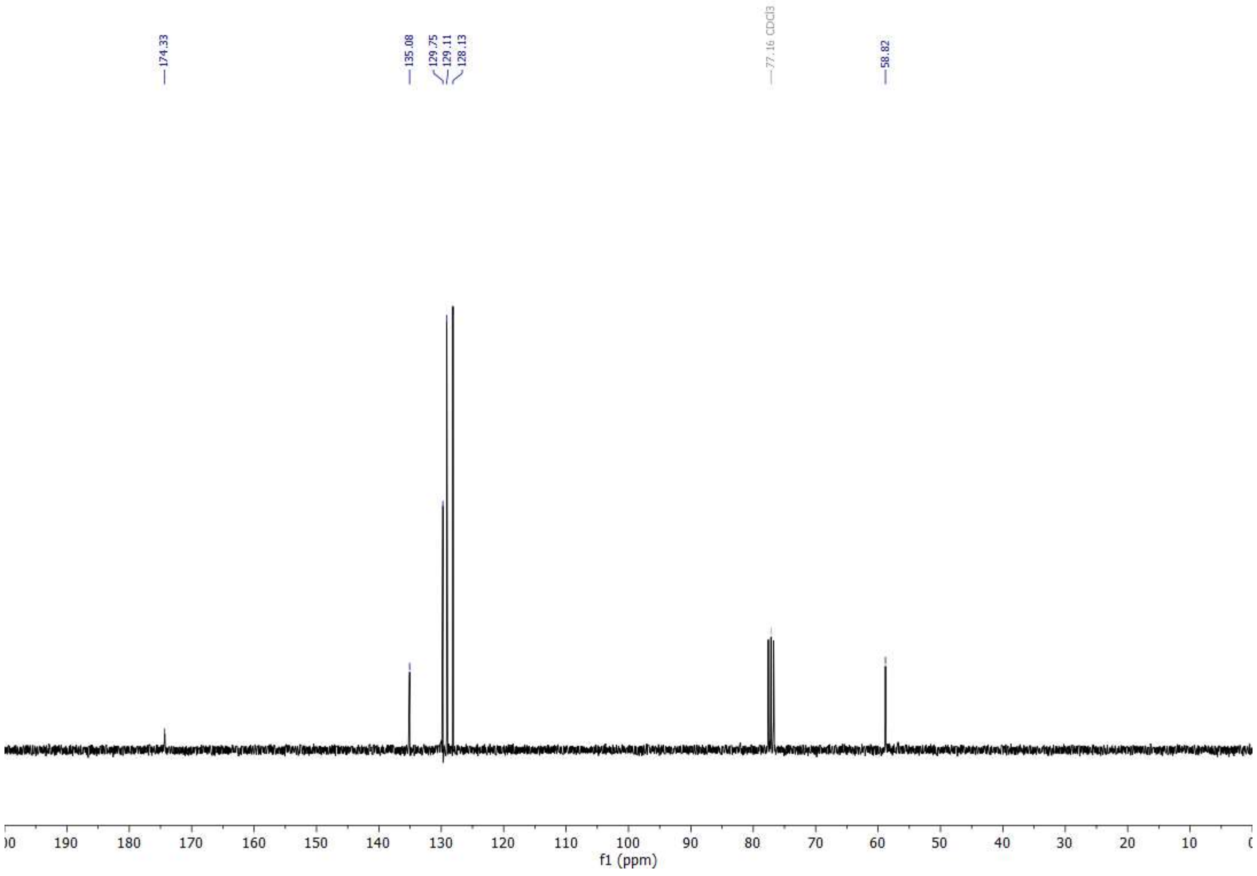

174.33
135.08
129.75
129.11
128.13
77.16 CDCl3
58.82
f1 (ppm)

***2-Chloro-2-(4-nitrophenyl)acetic acid (2a)***

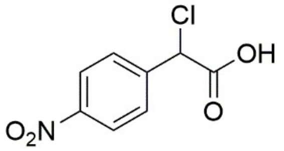

crude

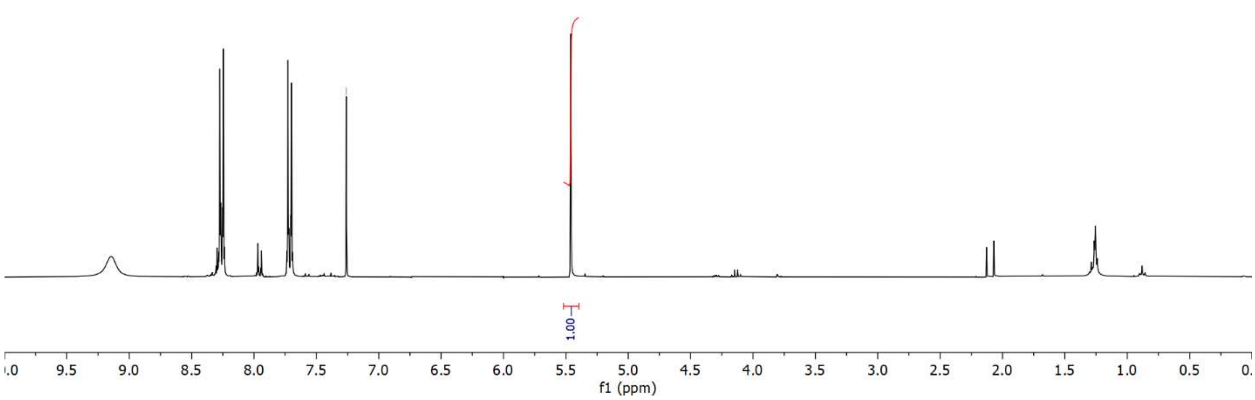


purified

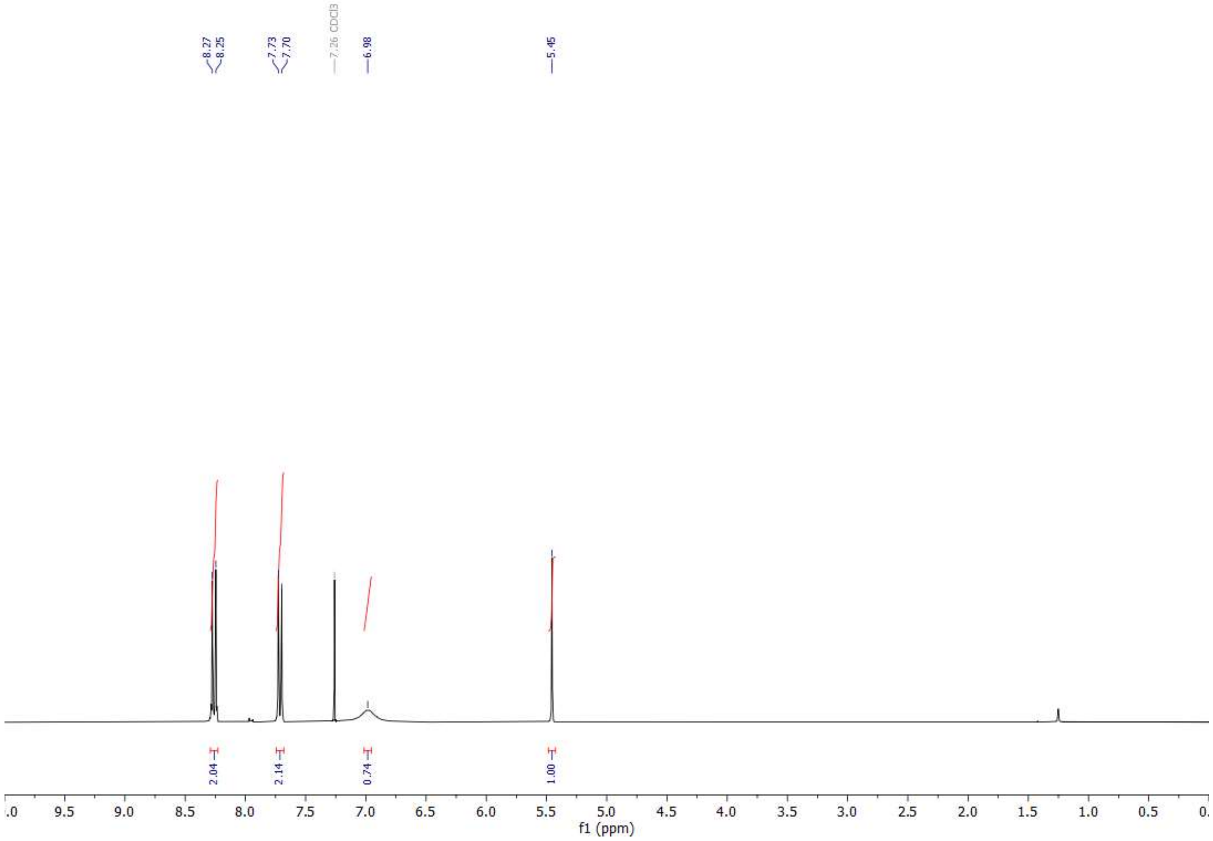

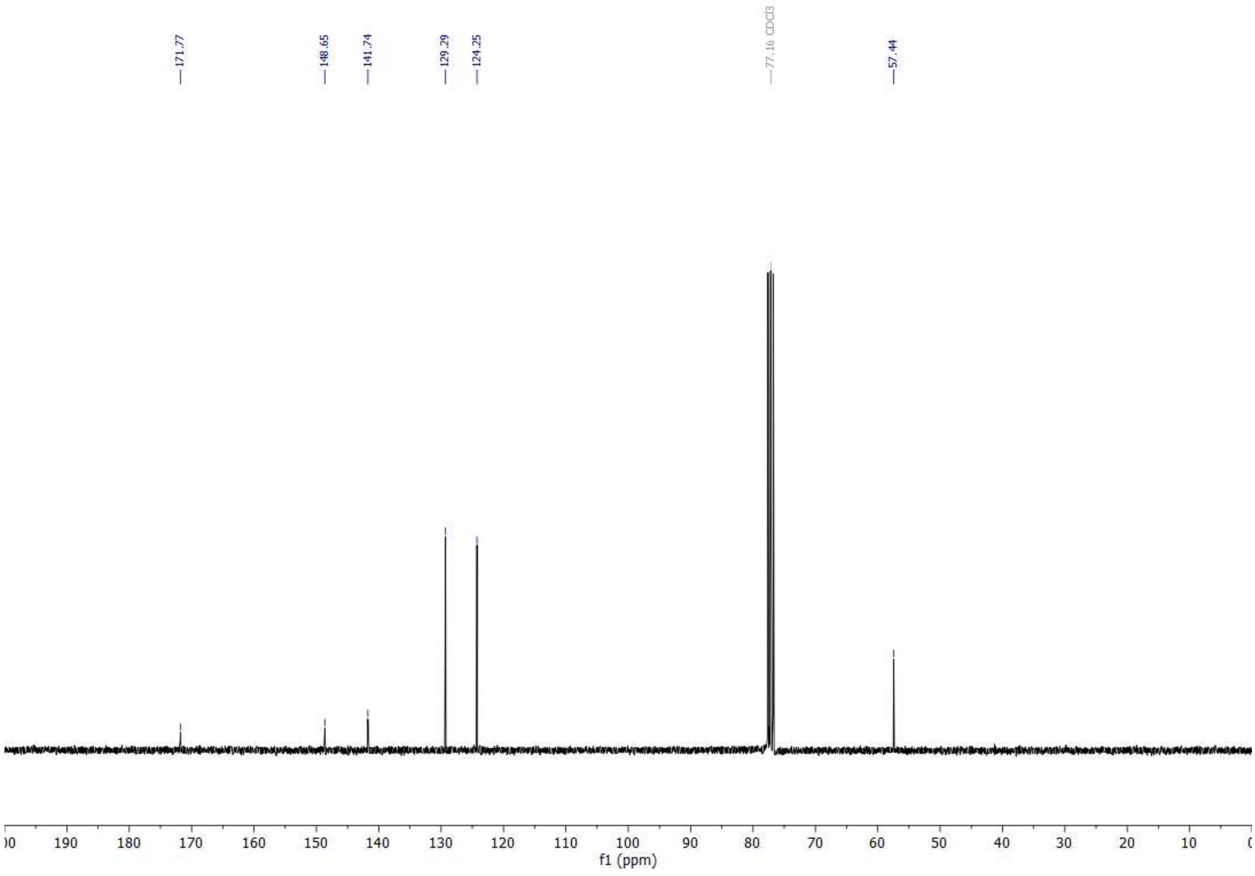

171.77
148.65
141.74
129.29
124.25
77.16 CDCl3
57.44
190 180 170 160 150 140 130 120 110 100 90 80 70 60 50 40 30 20 10
f1 (ppm)


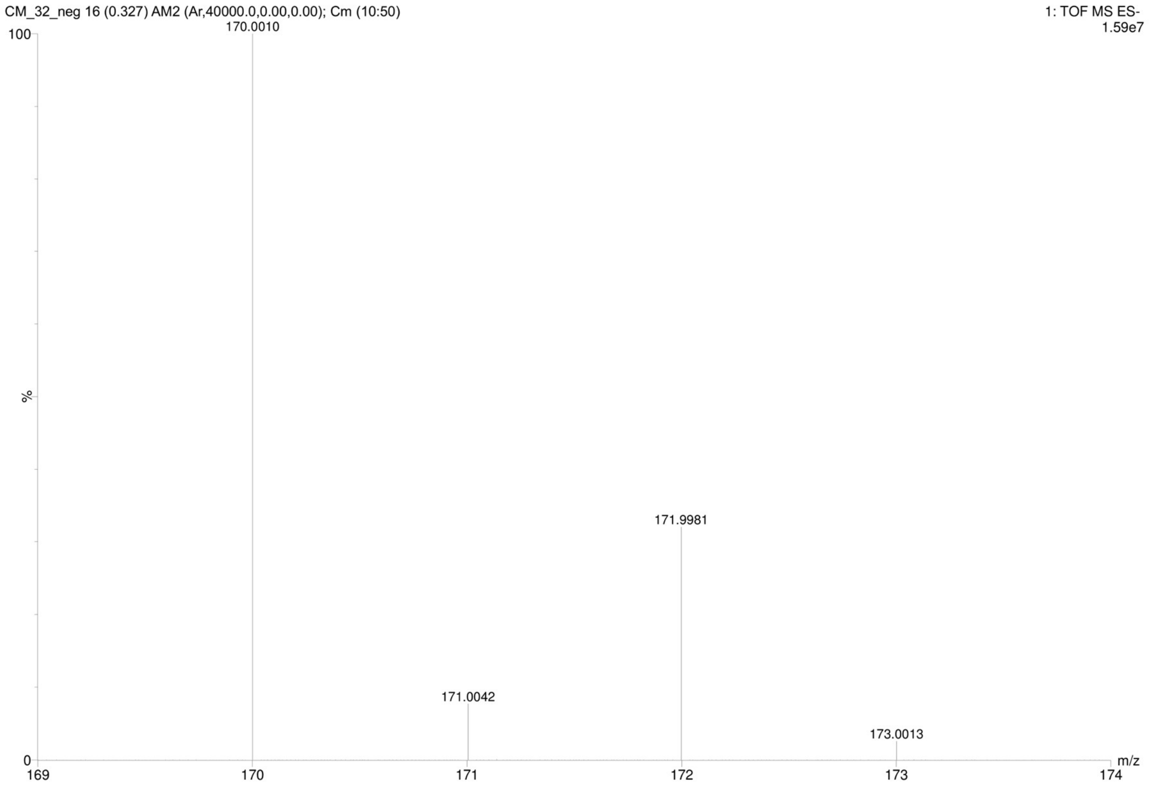

CM_32_neg 16 (0.327) AM2 (Ar,40000.0,0.00,0.00); Cm (10:50)
1: TOF MS ES-
1.59e7
100
%
0
170.0010
171.0042
171.9981
173.0013
169 170 171 172 173 174
m/z

***2-Chloro-2-(4-cyanophenyl)acetic acid (3a)*** crude

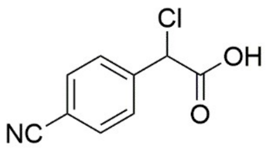

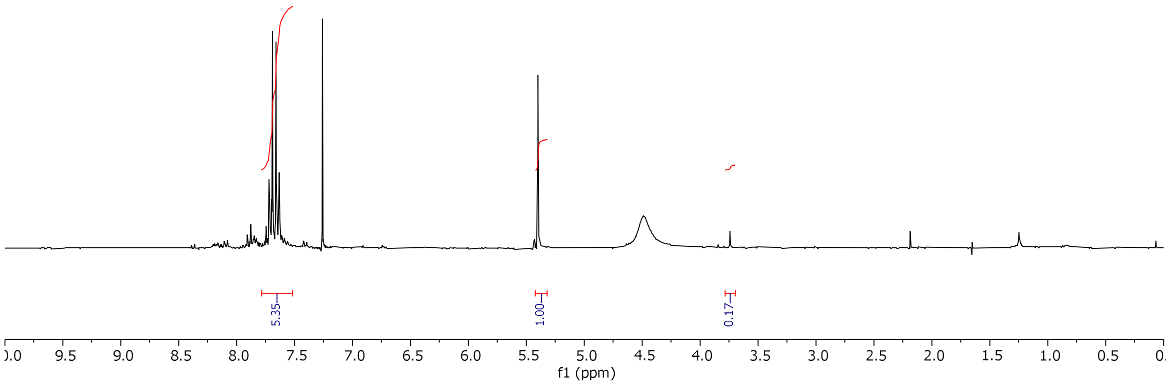

***2-Chloro-2-(4-(trifluoromethyl)phenyl)acetic acid (4a)***

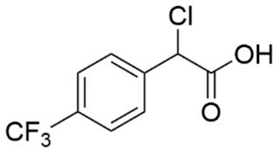

crude

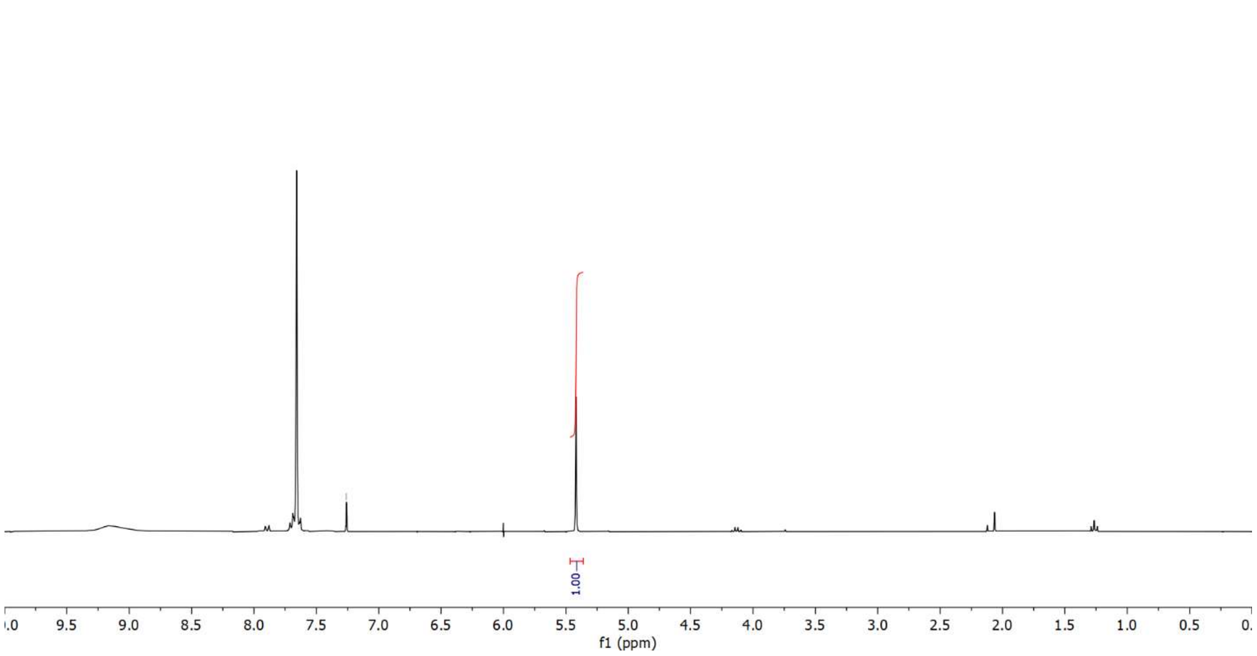


purified

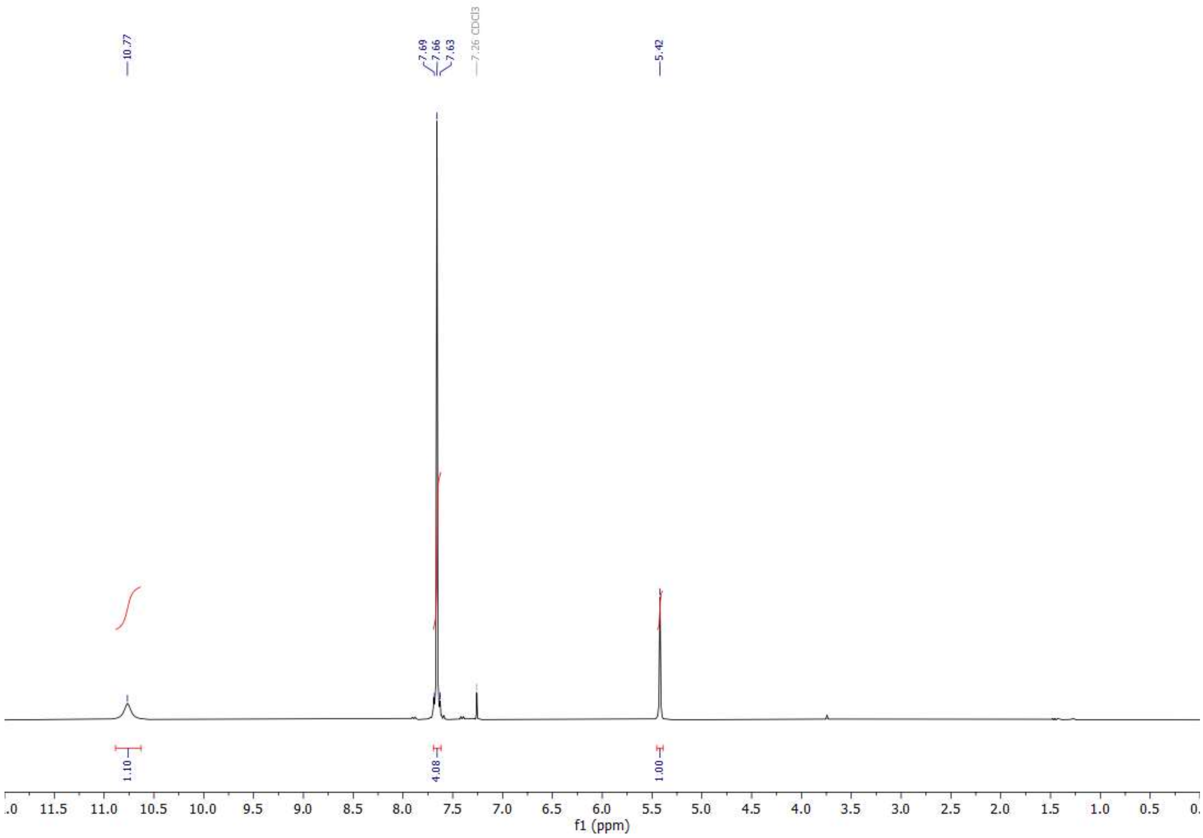

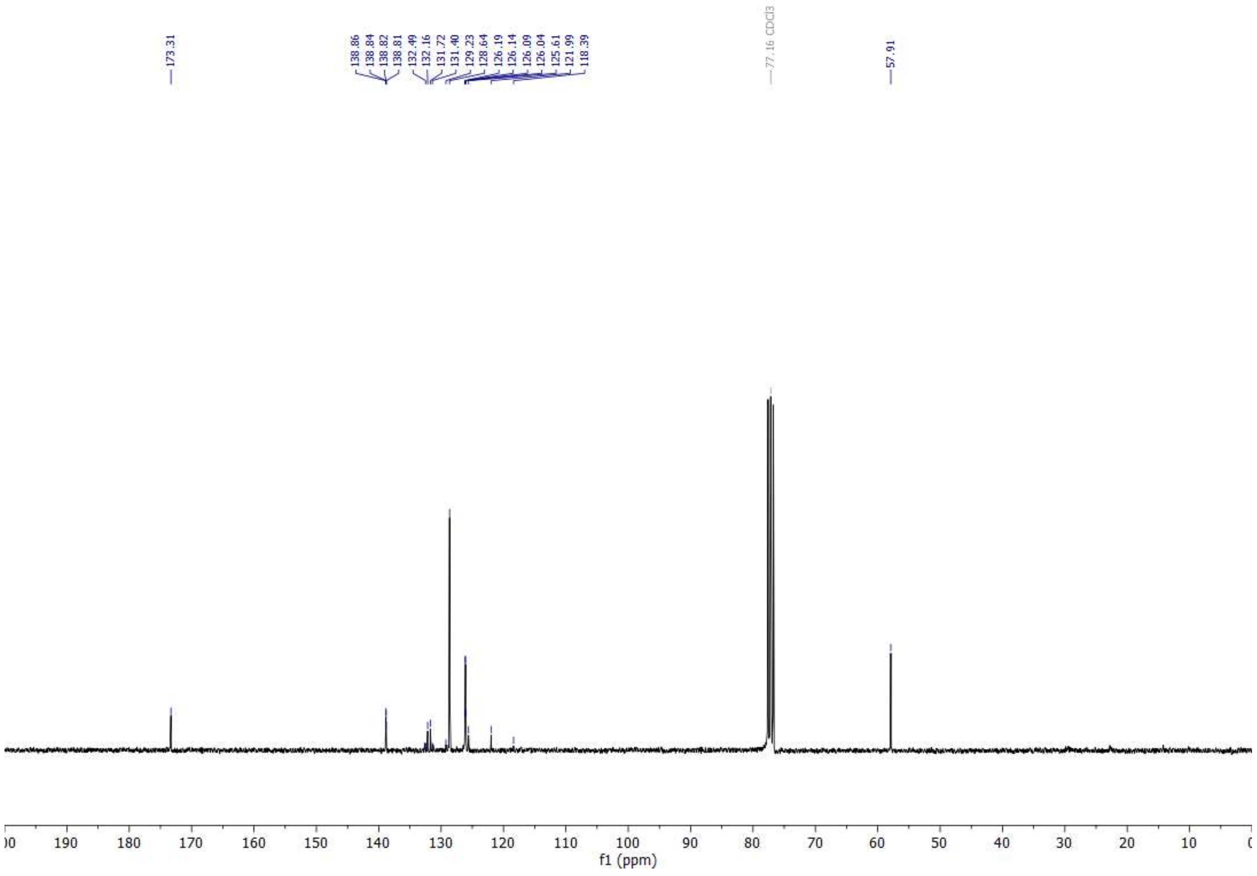
173.31
138.86
138.84
138.82
138.81
132.49
132.16
131.72
131.40
129.23
128.64
126.19
126.14
126.09
126.04
125.61
121.99
118.39
77.16 CDCl3
57.91
f1 (ppm)

***2-Chloro-2-(4-(methoxycarbonyl)phenyl)acetic acid (5a)***

crude

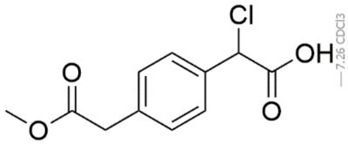


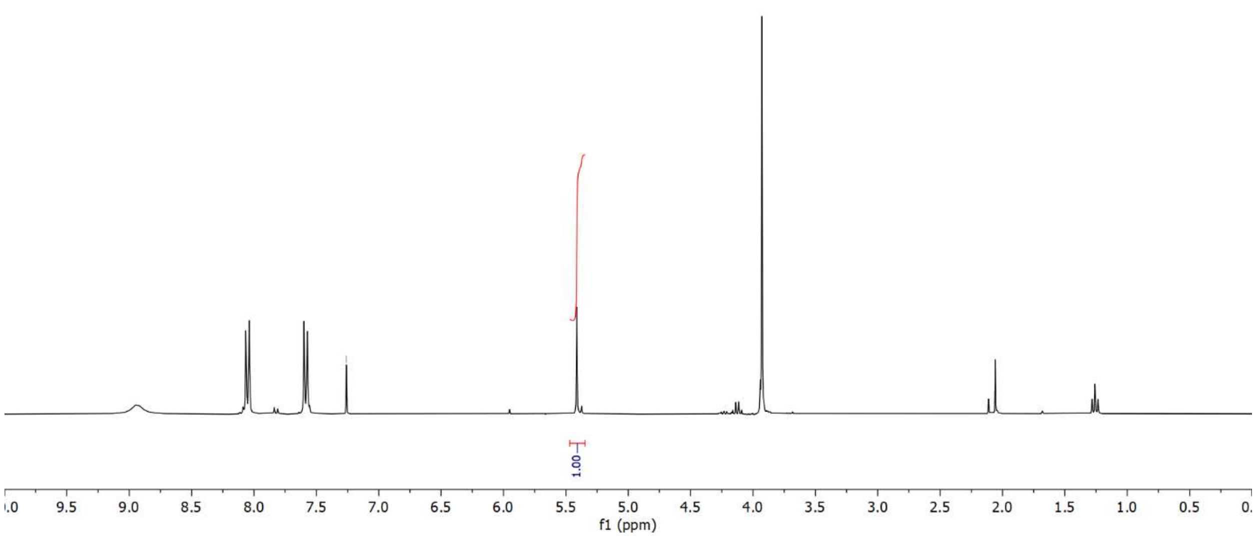


purified

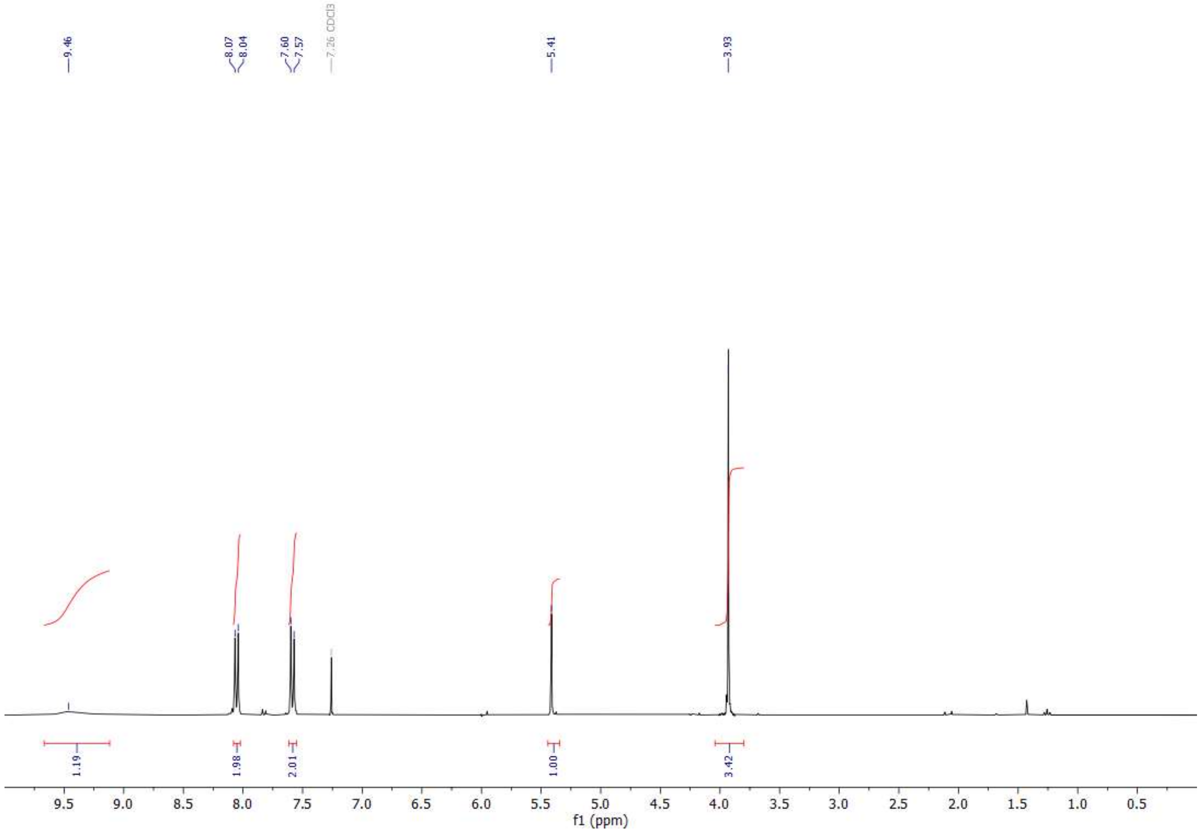

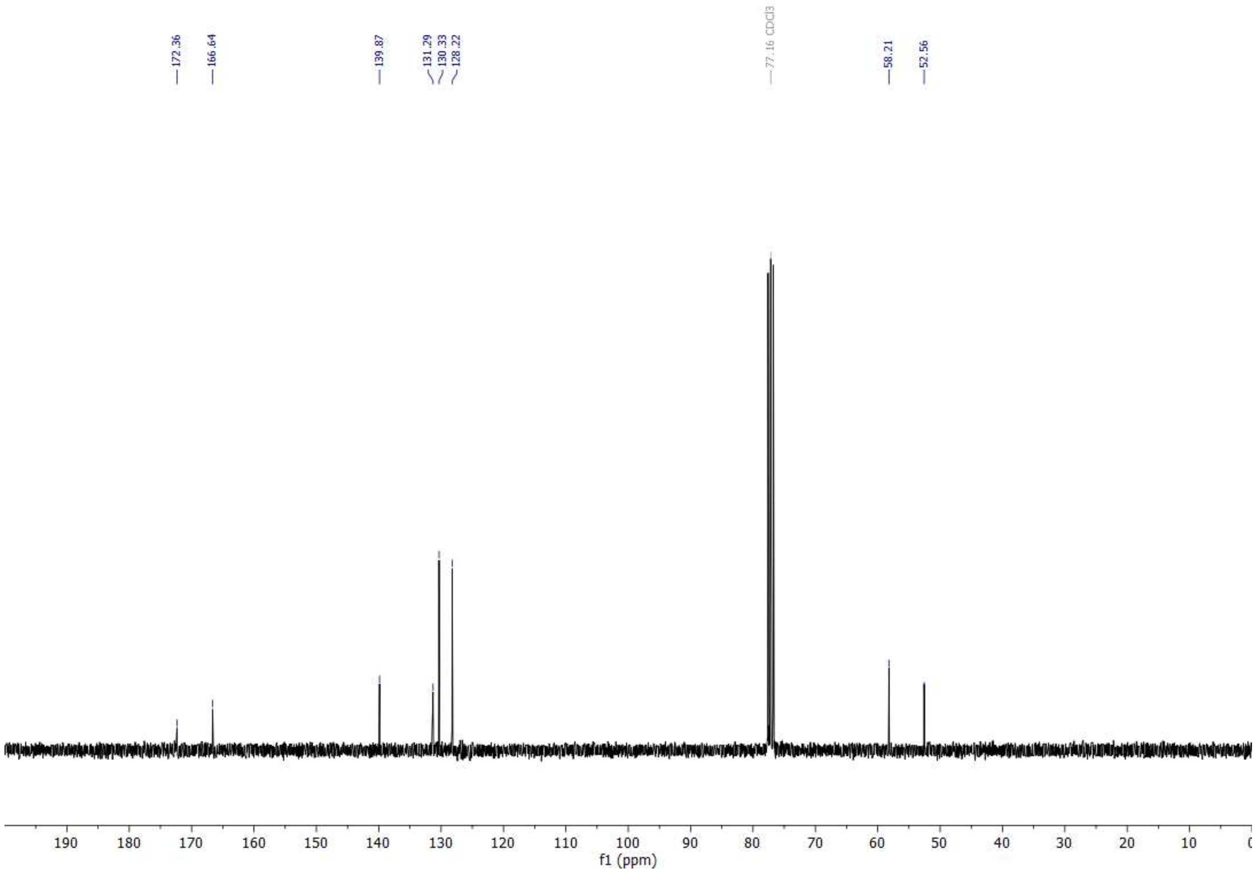
172.36
166.64
139.87
131.29
130.33
128.22
77.16 CDCl3
58.21
52.56
190
180
170
160
150
140
130
120
110
100
90
80
70
60
50
40
30
20
10
f1 (ppm)

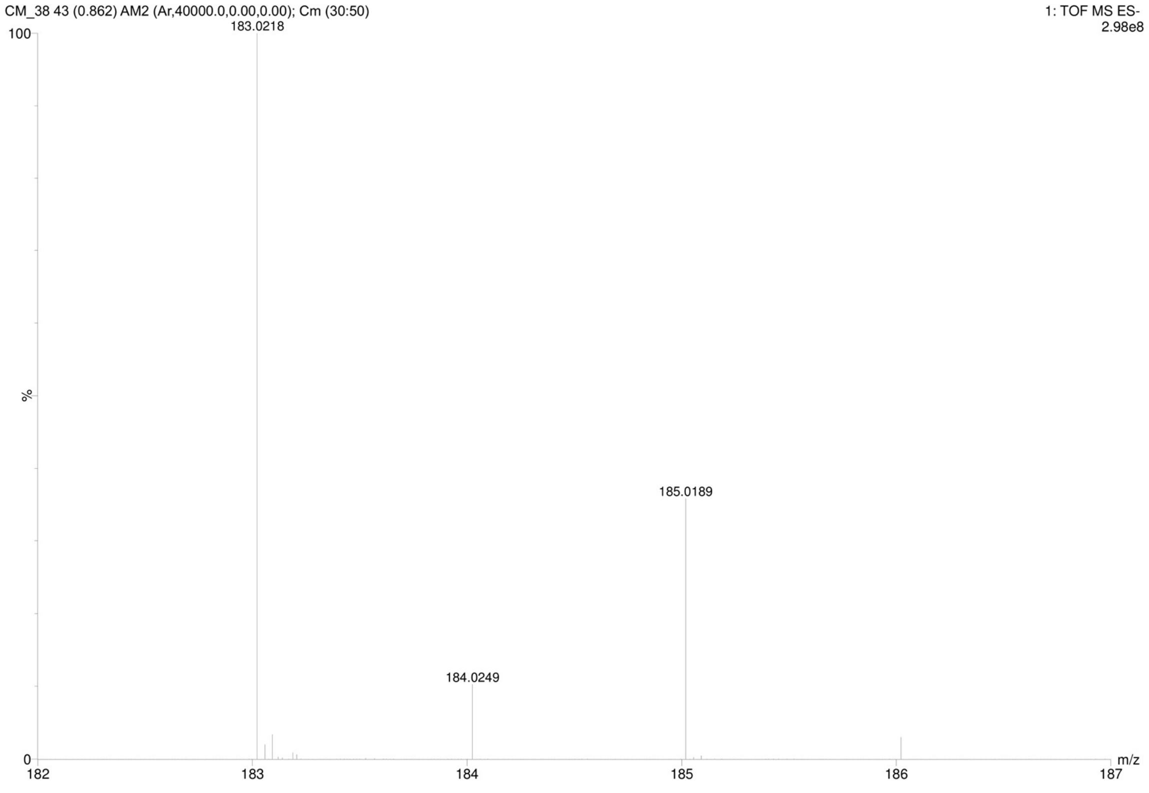
CM_38 43 (0.862) AM2 (Ar,40000.0,0.00,0.00); Cm (30:50)
1: TOF MS ES-
2.98e8
183.0218
185.0189
184.0249
100
%
0
182
183
184
185
186
187
m/z

***2-Chloro-2-(4-fluorophenyl)acetic acid (6a)***

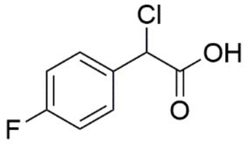


crude

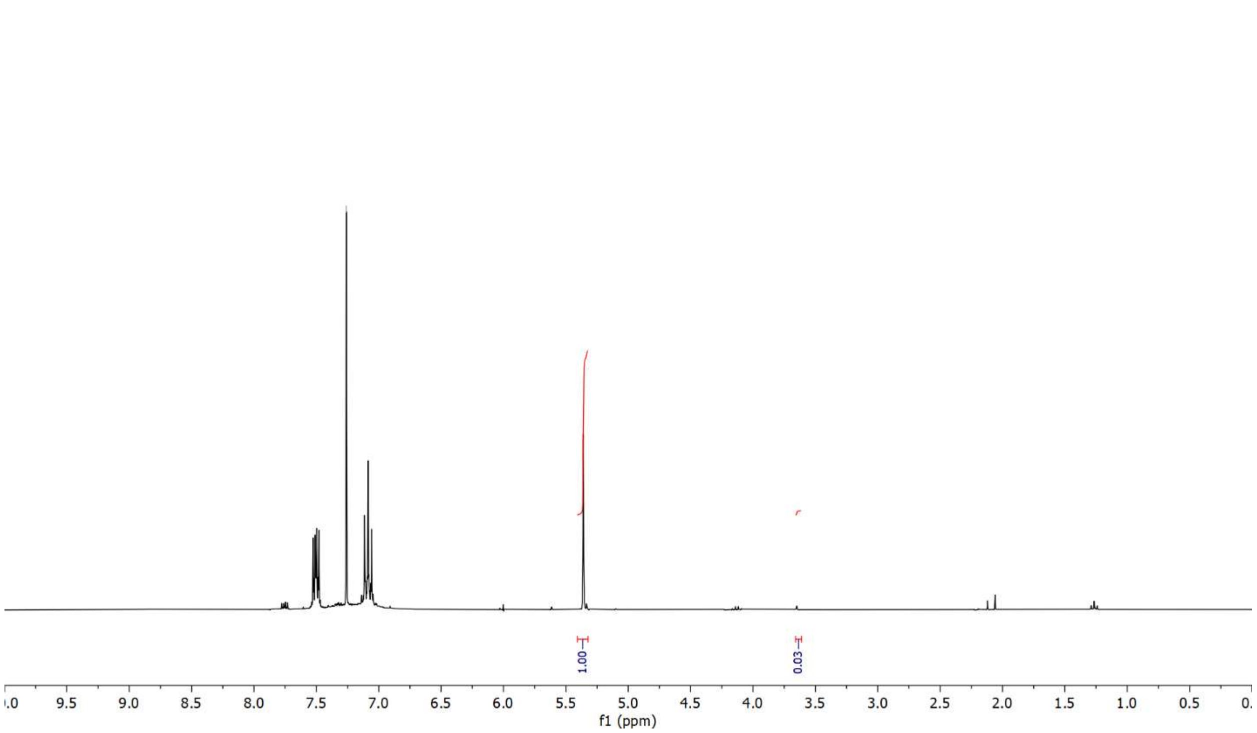


purified

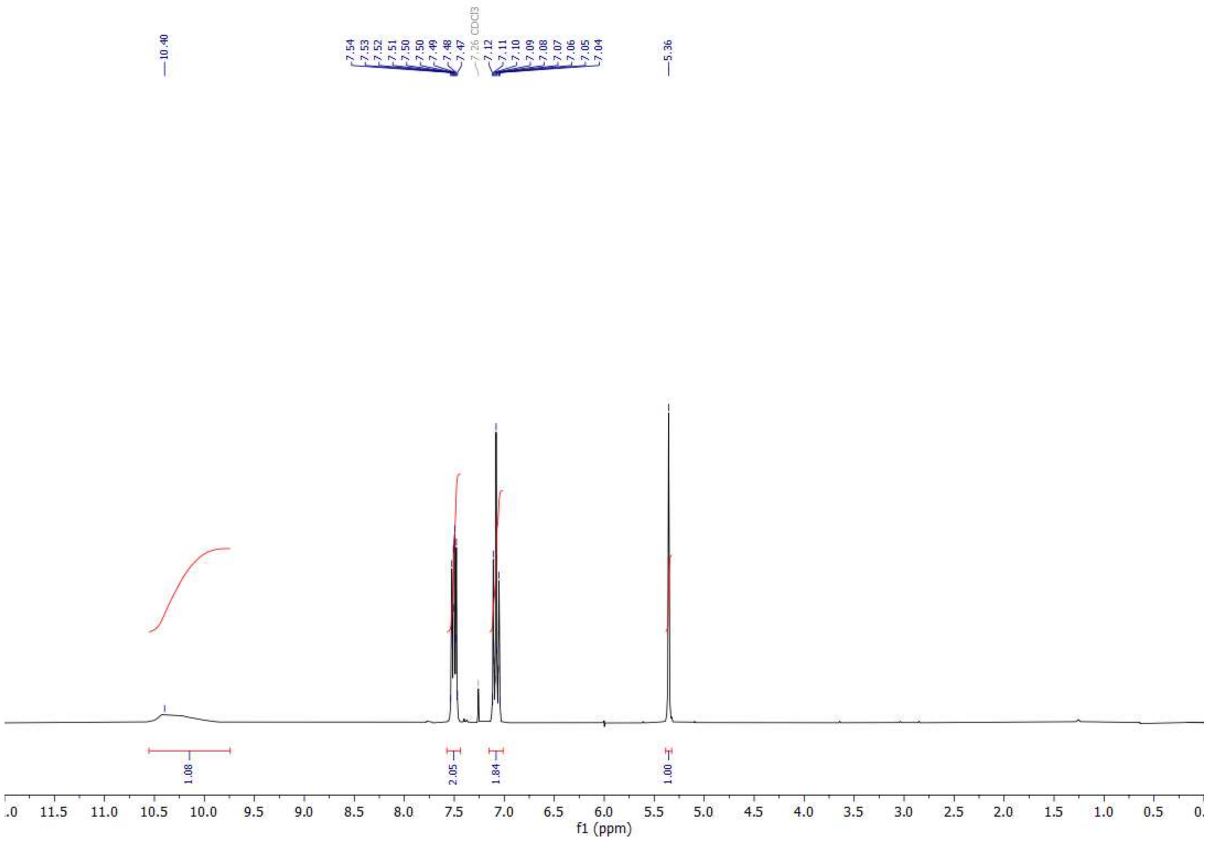

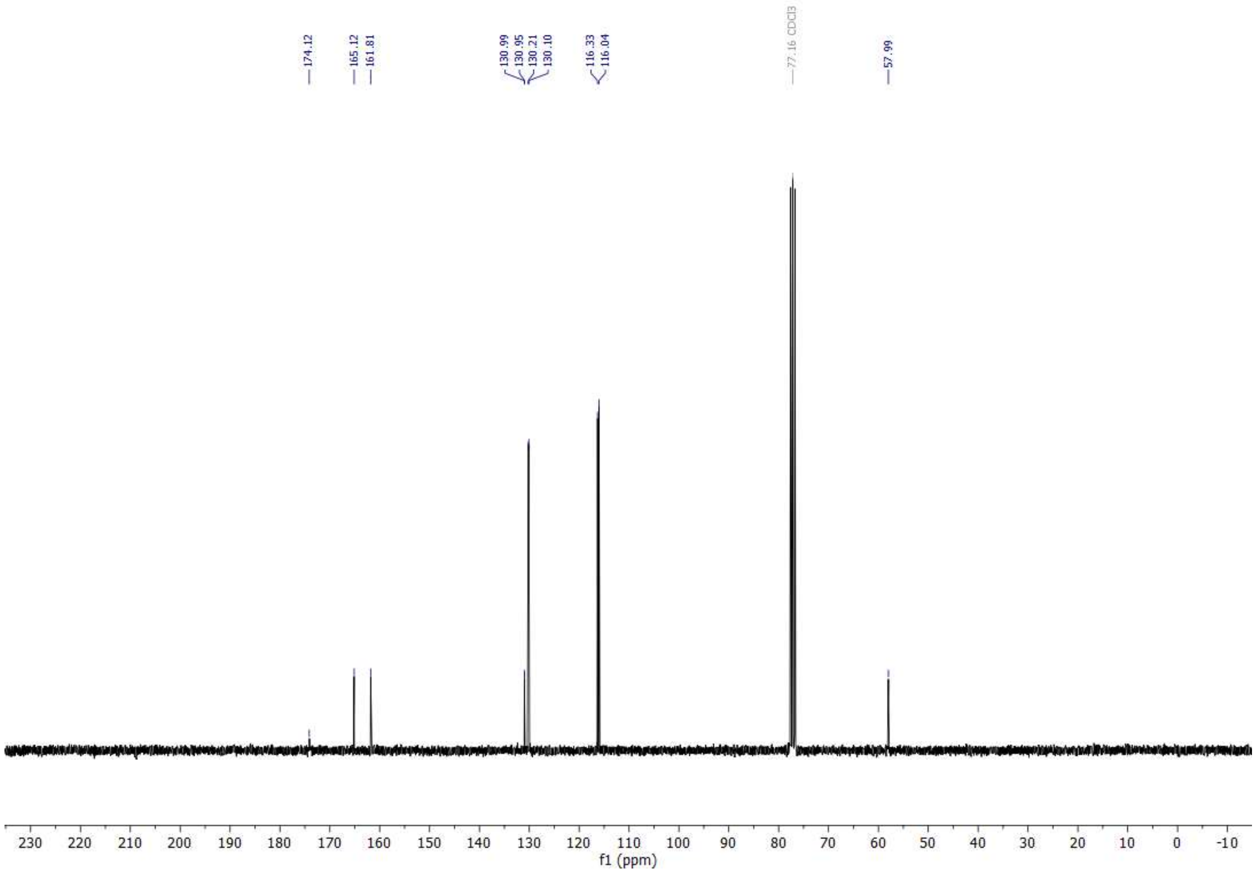

174.12
165.12
161.81
130.99
130.95
130.21
130.10
116.33
116.04
77.16 CDCl3
57.99
230 220 210 200 190 180 170 160 150 140 130 120 110 100 90 80 70 60 50 40 30 20 10 0 -10
f1 (ppm)

***2-Chloro-2-(4-chlorophenyl)acetic acid (7a)***

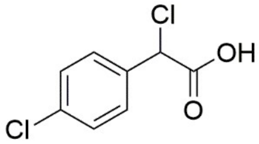

crude

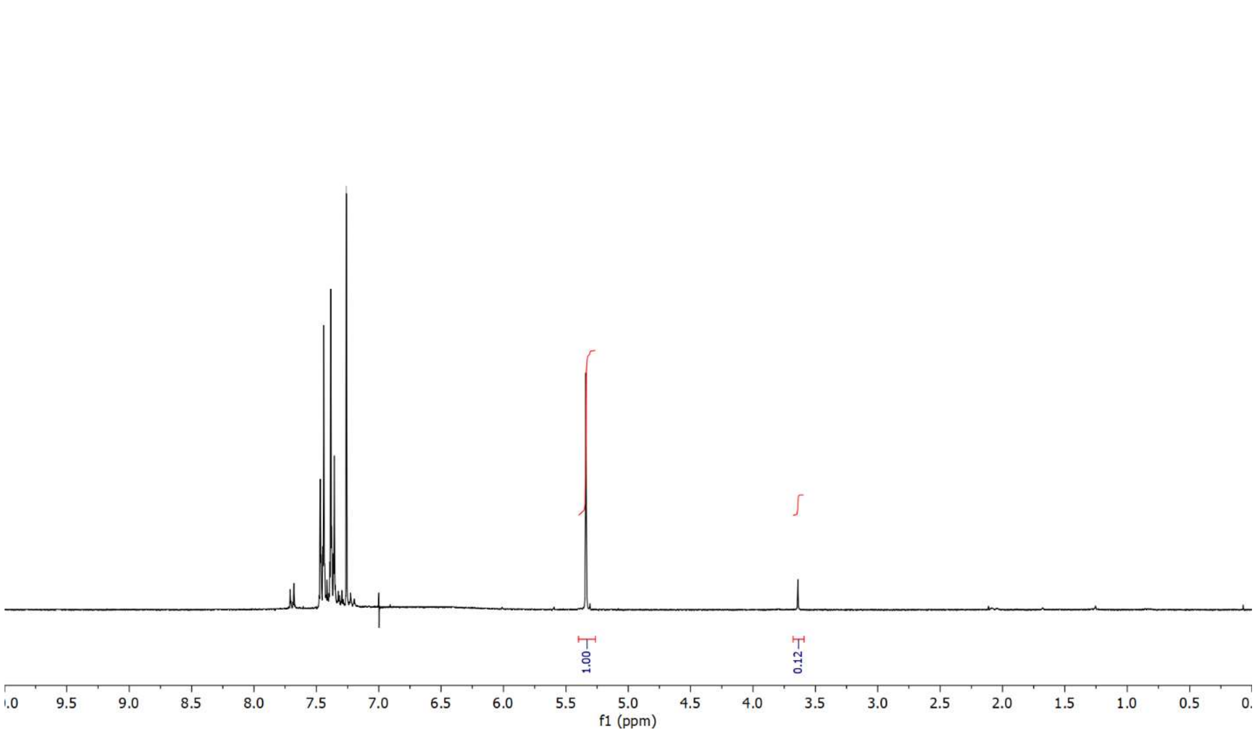


purified

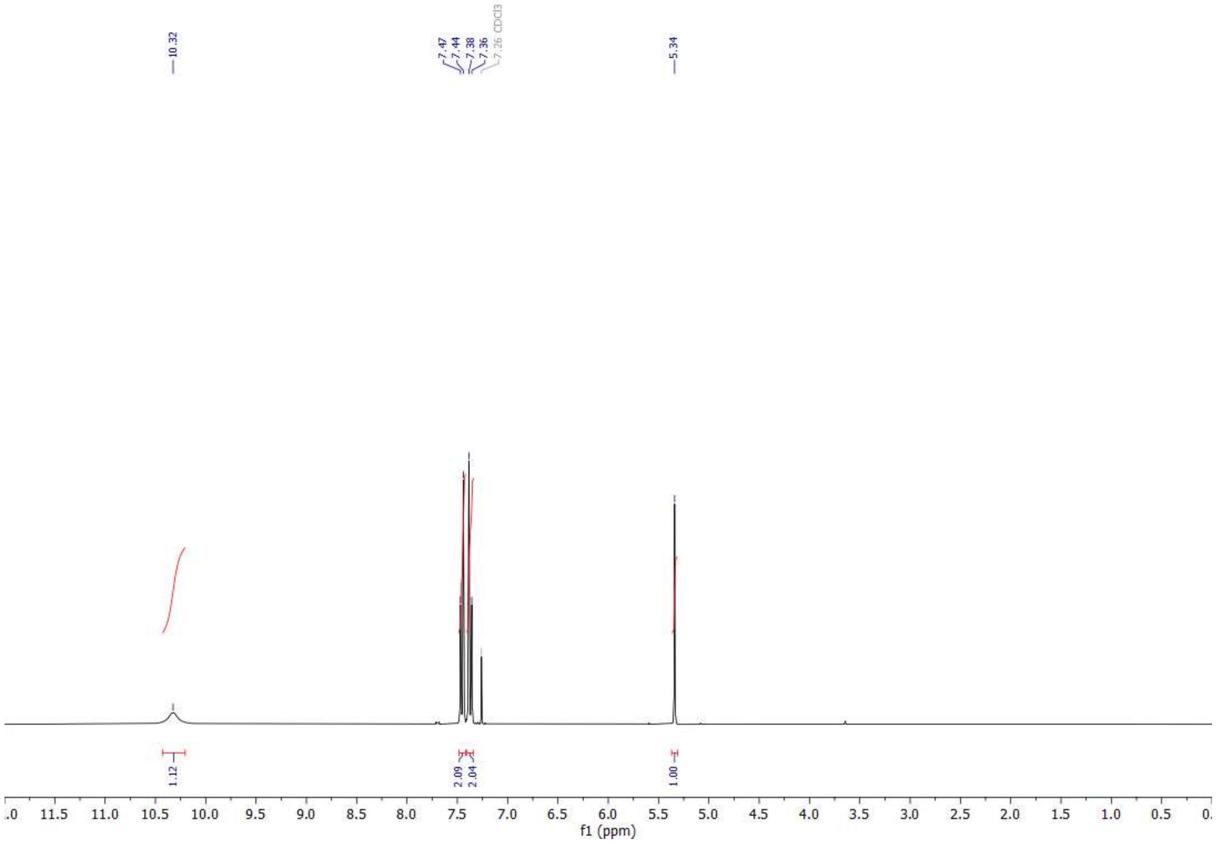

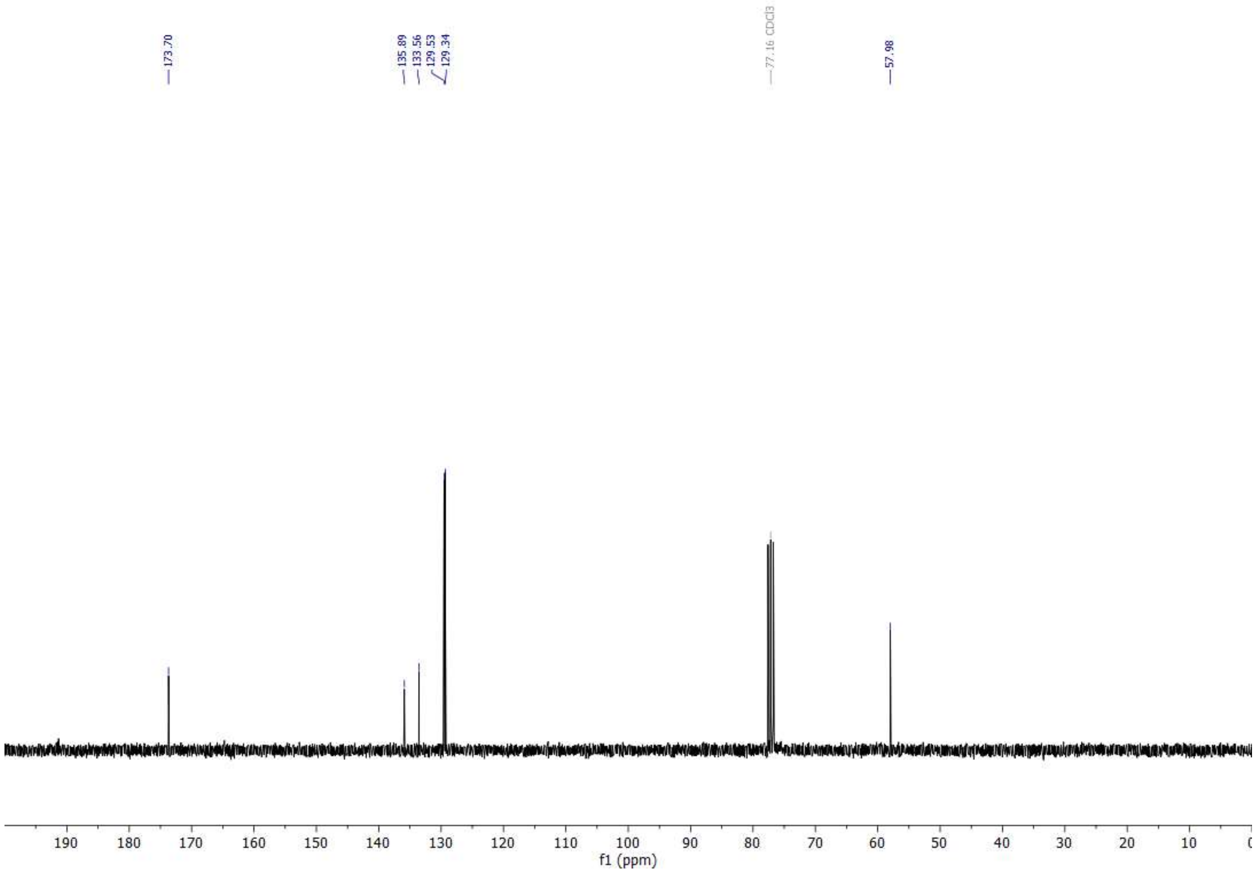
173.70
135.89
133.56
129.53
129.34
77.16 CDCl3
57.98
f1 (ppm)

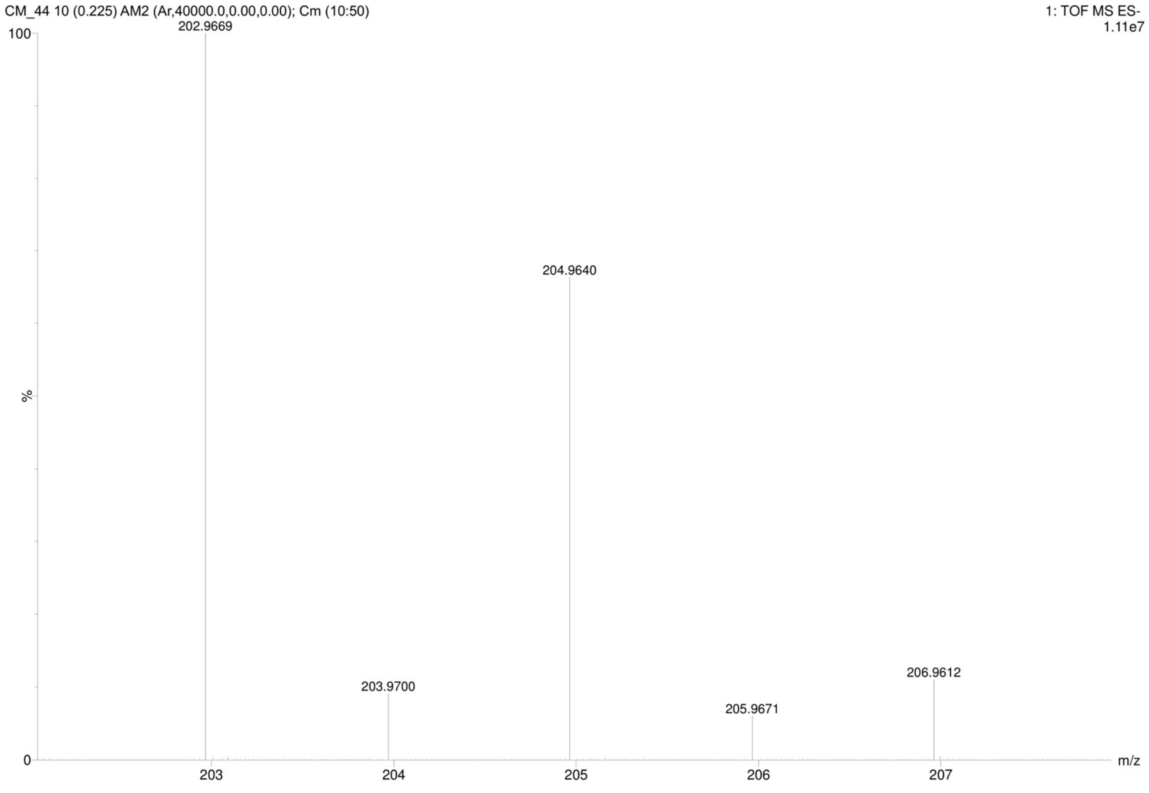
CM_44 10 (0.225) AM2 (Ar,40000.0,0.00,0.00); Cm (10:50)
1: TOF MS ES-
1.11e7
202.9669
204.9640
203.9700
205.9671
206.9612
%
m/z

***2-Chloro-2-(4-bromophenyl)acetic acid (8a)***

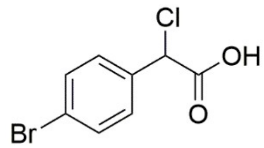


crude

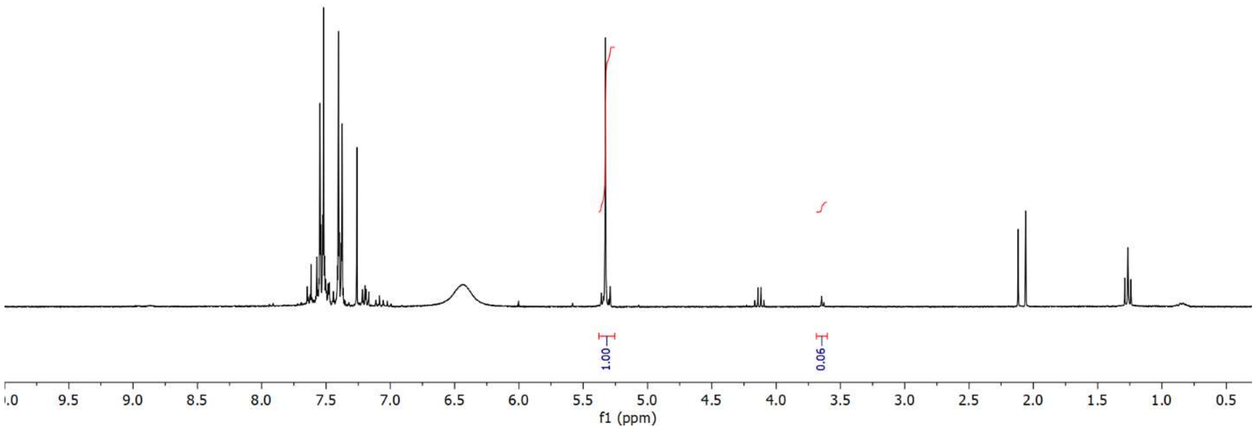


purified

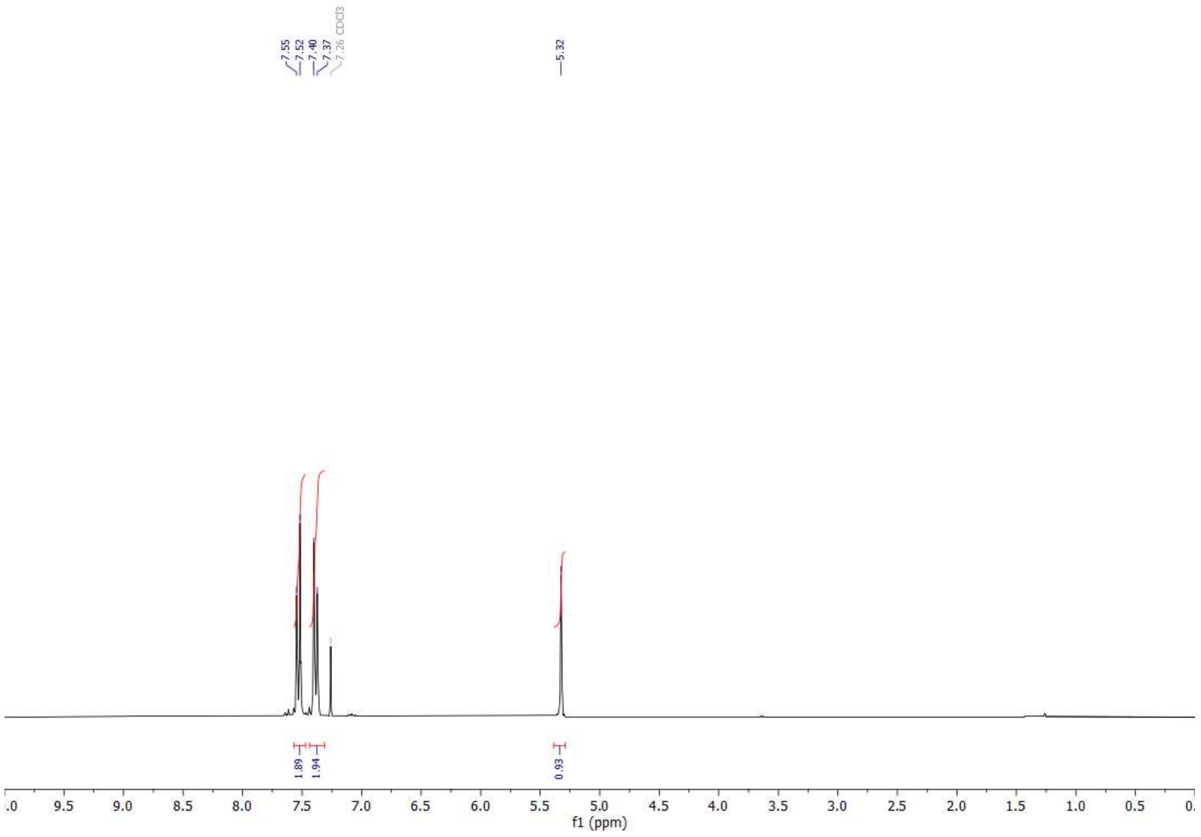

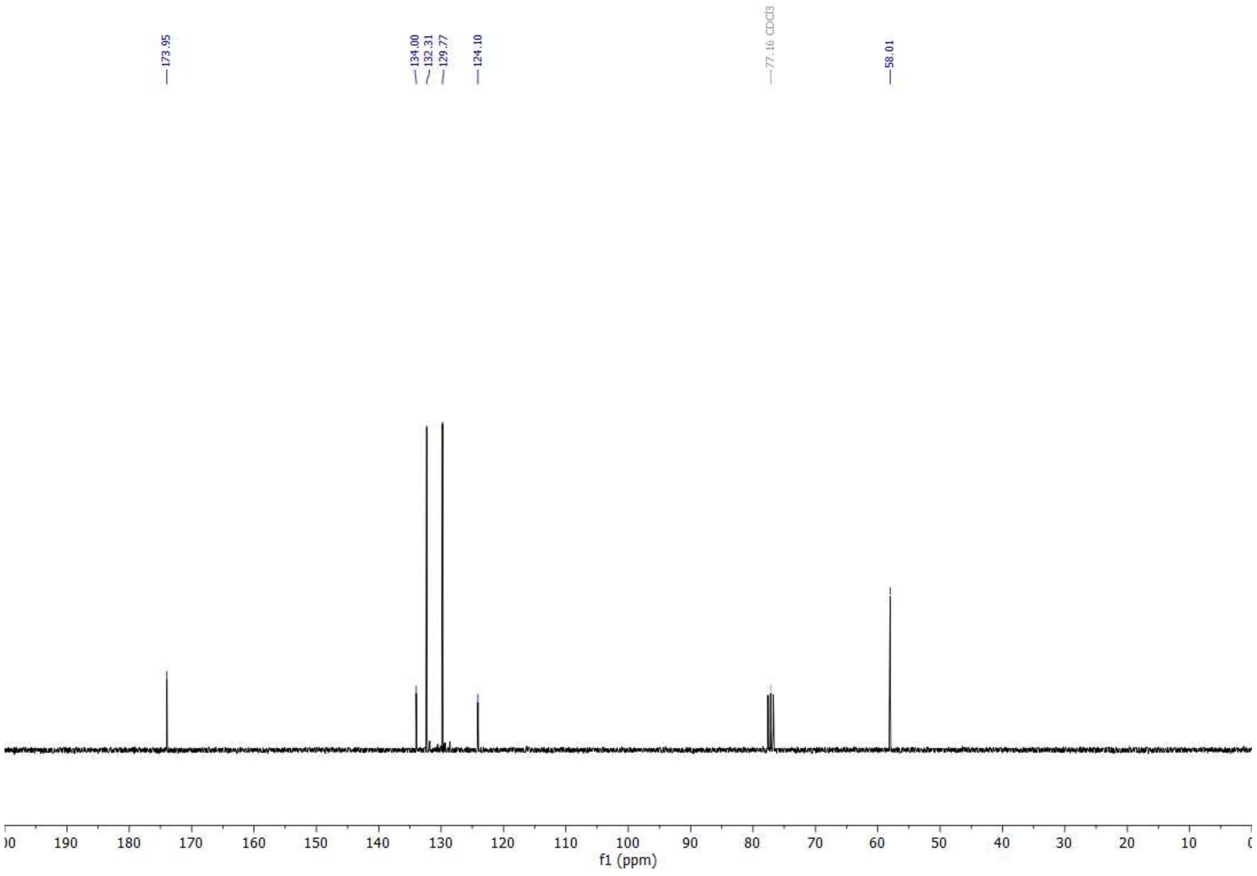

173.95
134.00
132.31
129.77
124.10
77.16 CDCl3
58.01
190
180
170
160
150
140
130
120
110
100
90
80
70
60
50
40
30
20
10
f1 (ppm)

***2-Chloro-2-(4-iodophenyl)acetic acid (9a)***

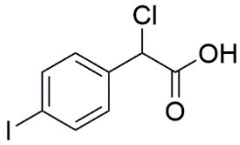

crude

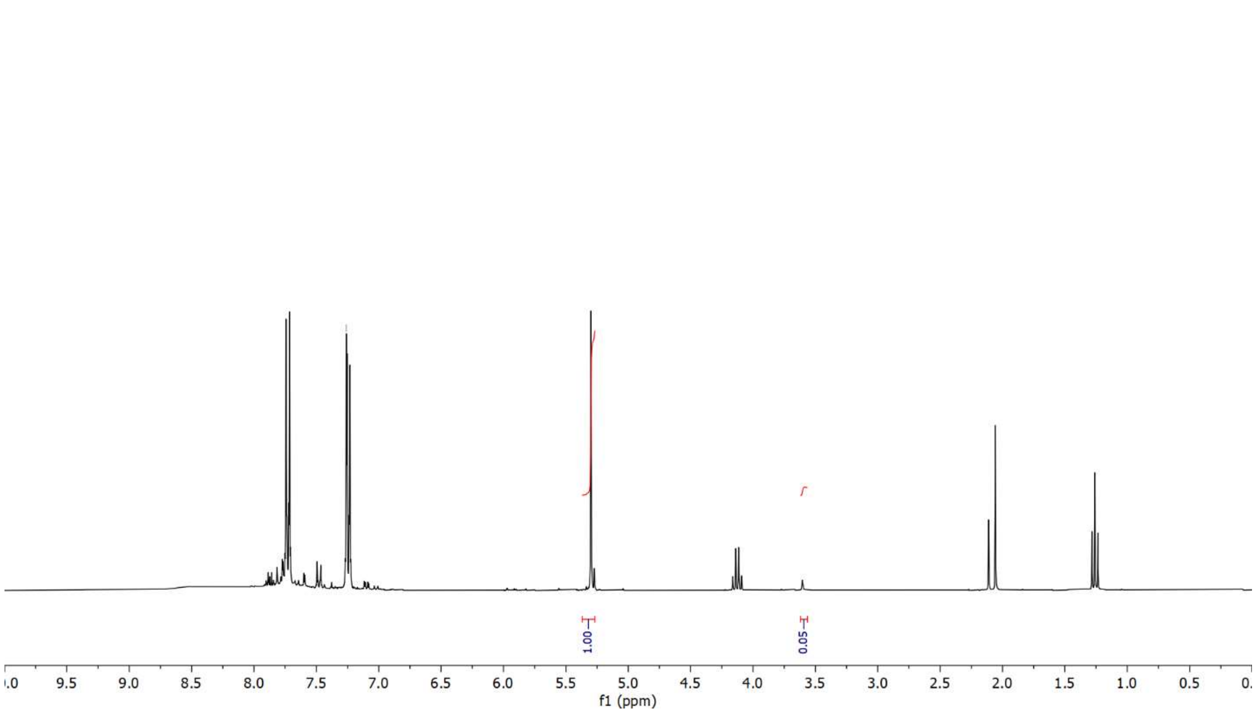


purified

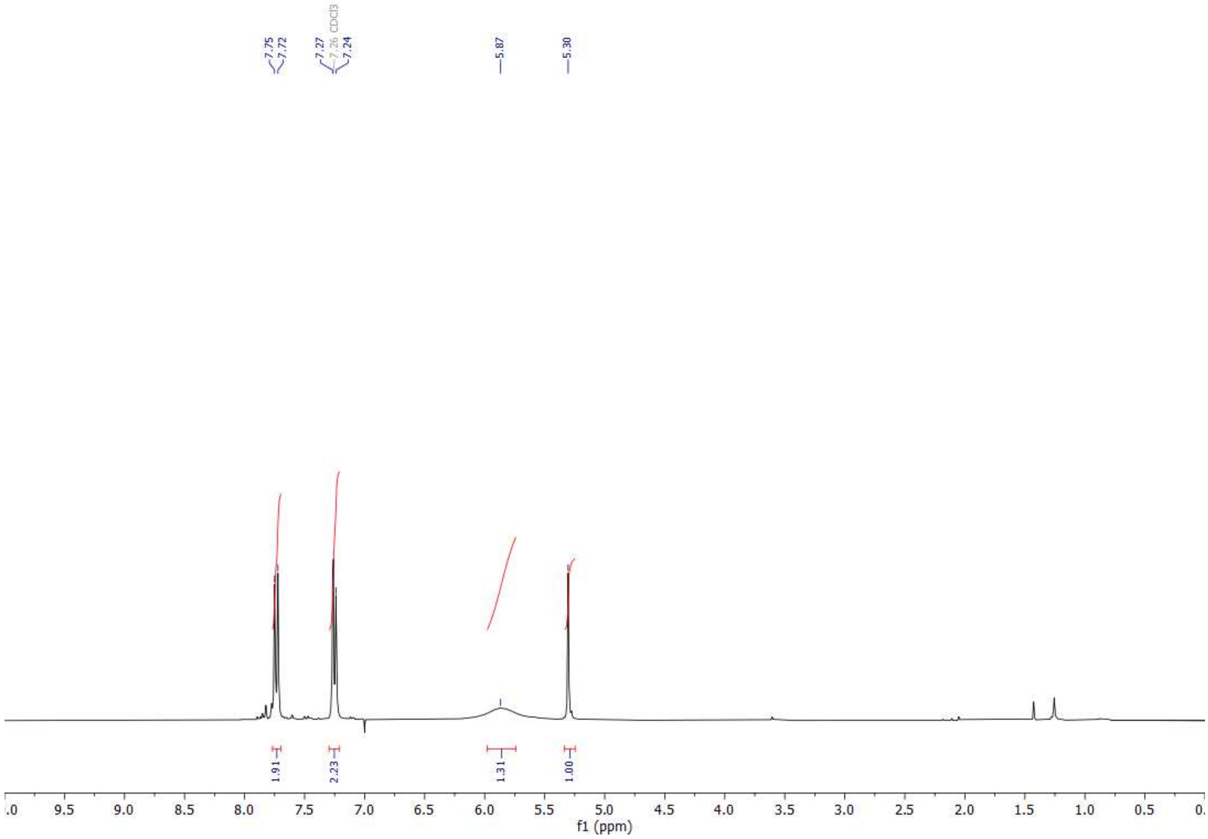

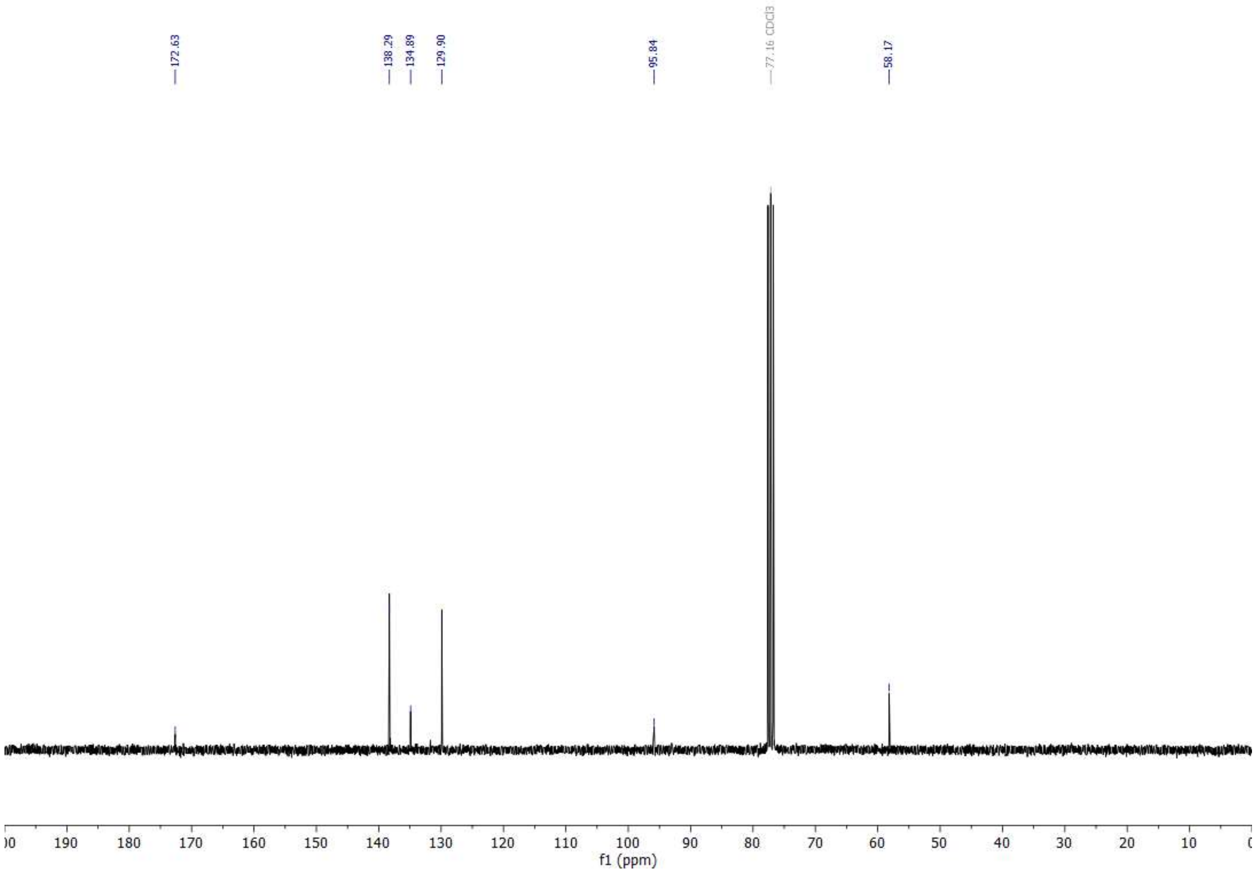
172.63
138.29
134.89
129.90
95.84
77.16 CDCl3
58.17
f1 (ppm)

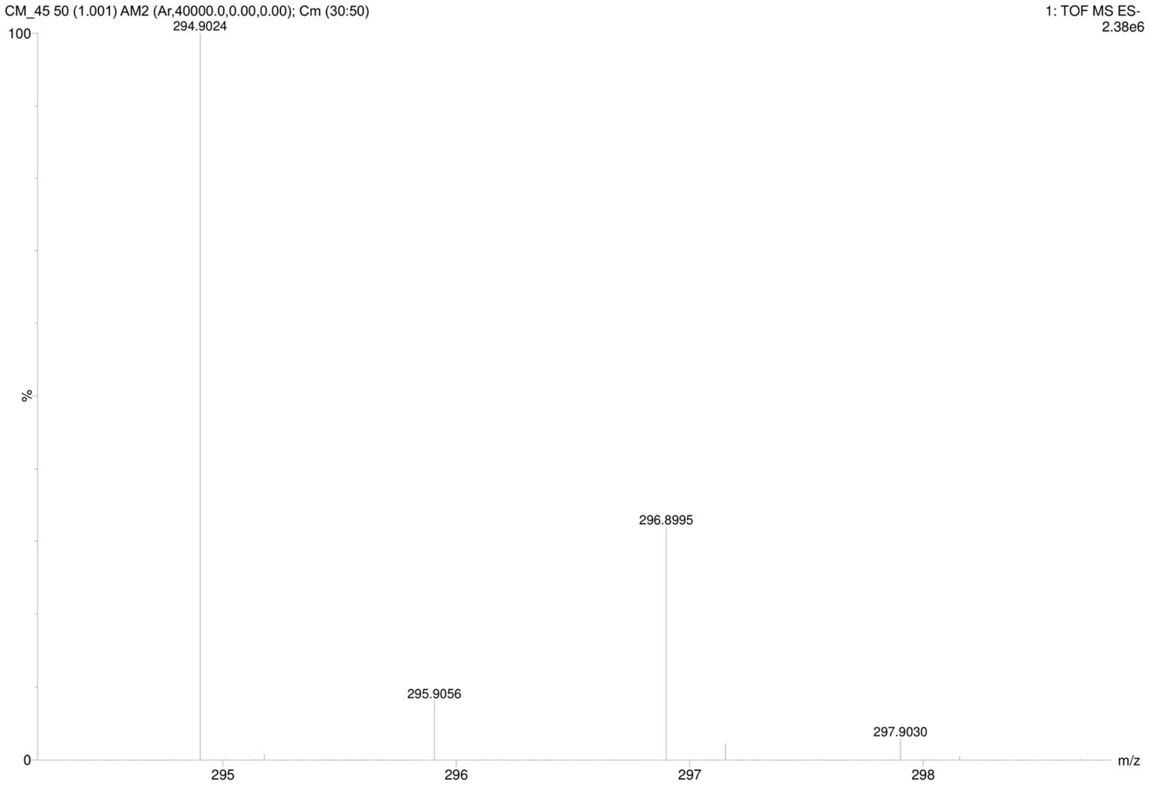
CM_45 50 (1.001) AM2 (Ar,40000.0,0.00,0.00); Cm (30:50)
1: TOF MS ES-
2.38e6
294.9024
296.8995
295.9056
297.9030
%
m/z

***2-Chloro-2-(p-tolyl)acetic acid (10a)***

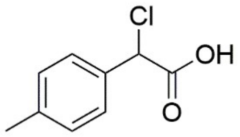


crude

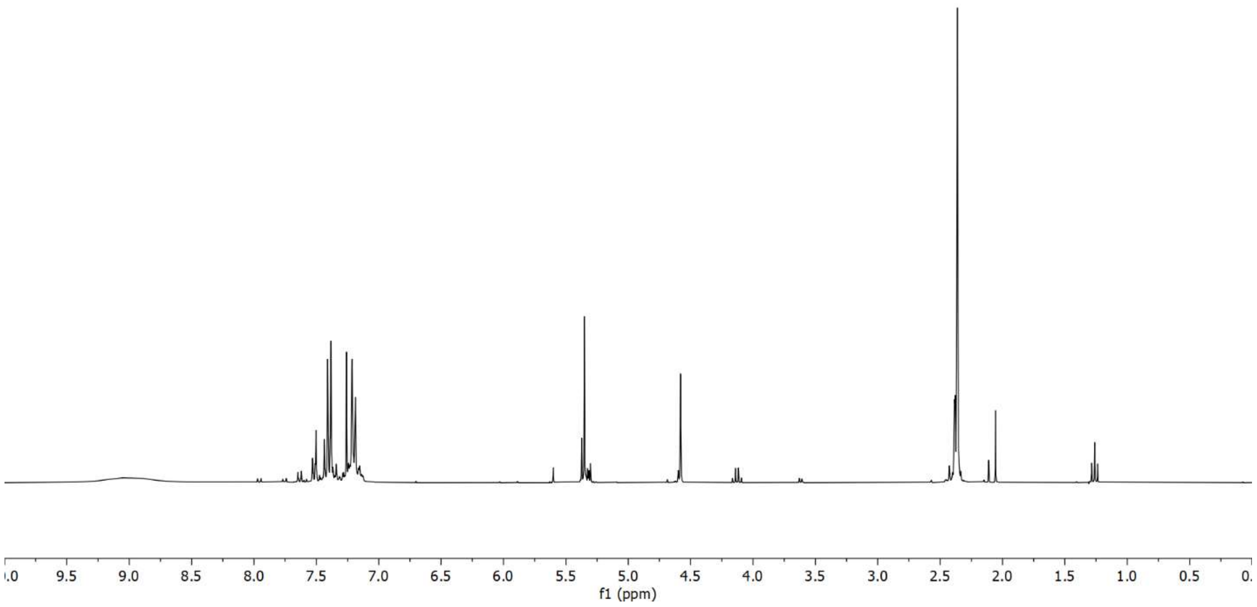


purified

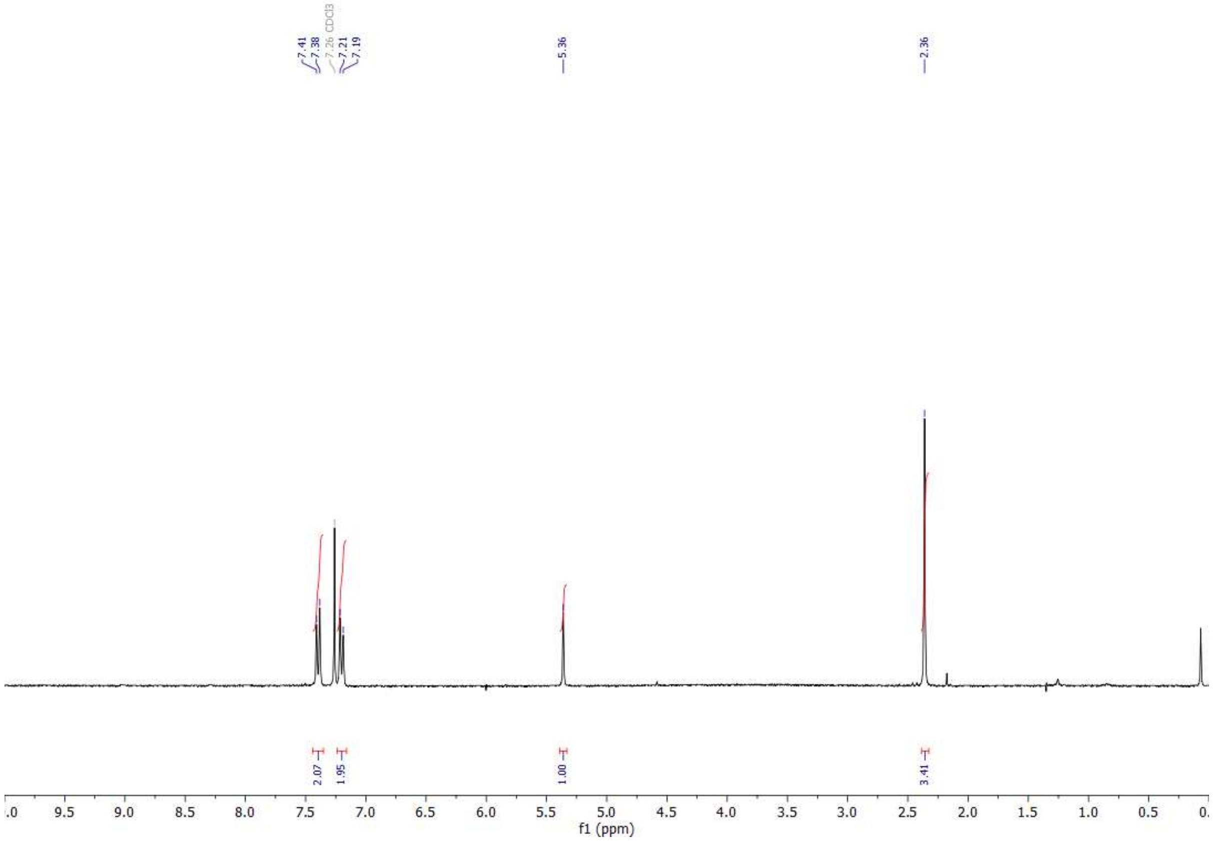

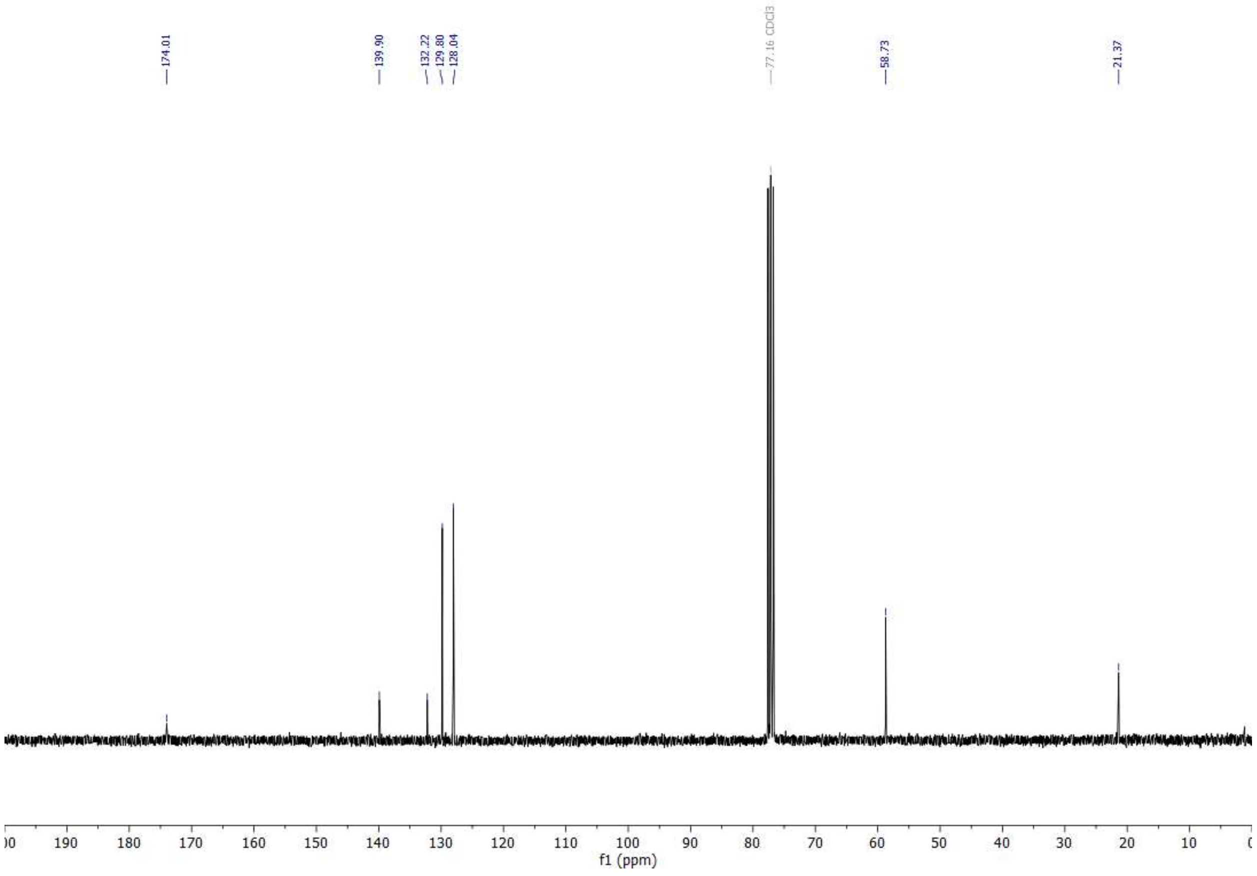
174.01
139.90
132.22
129.80
128.04
77.16 CDCl3
58.73
21.37
f1 (ppm)

***2-Chloro-2-(4-isopropylphenyl)acetic acid (11a)***

crude

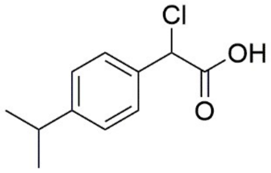


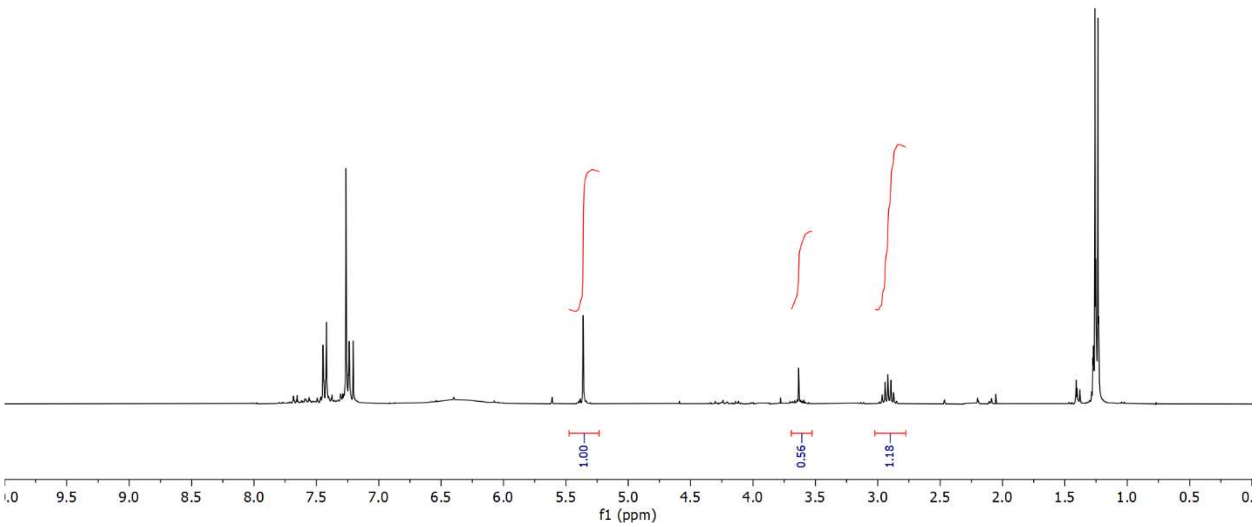


purified

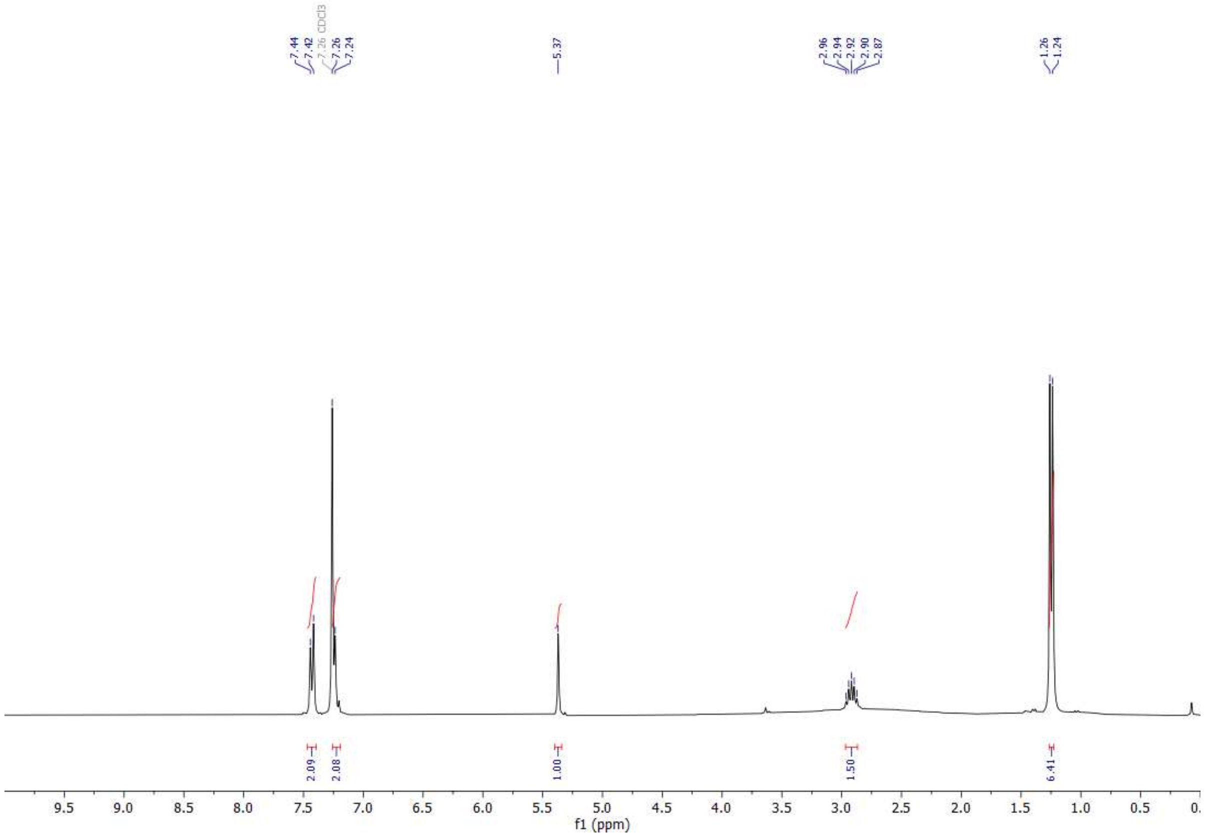

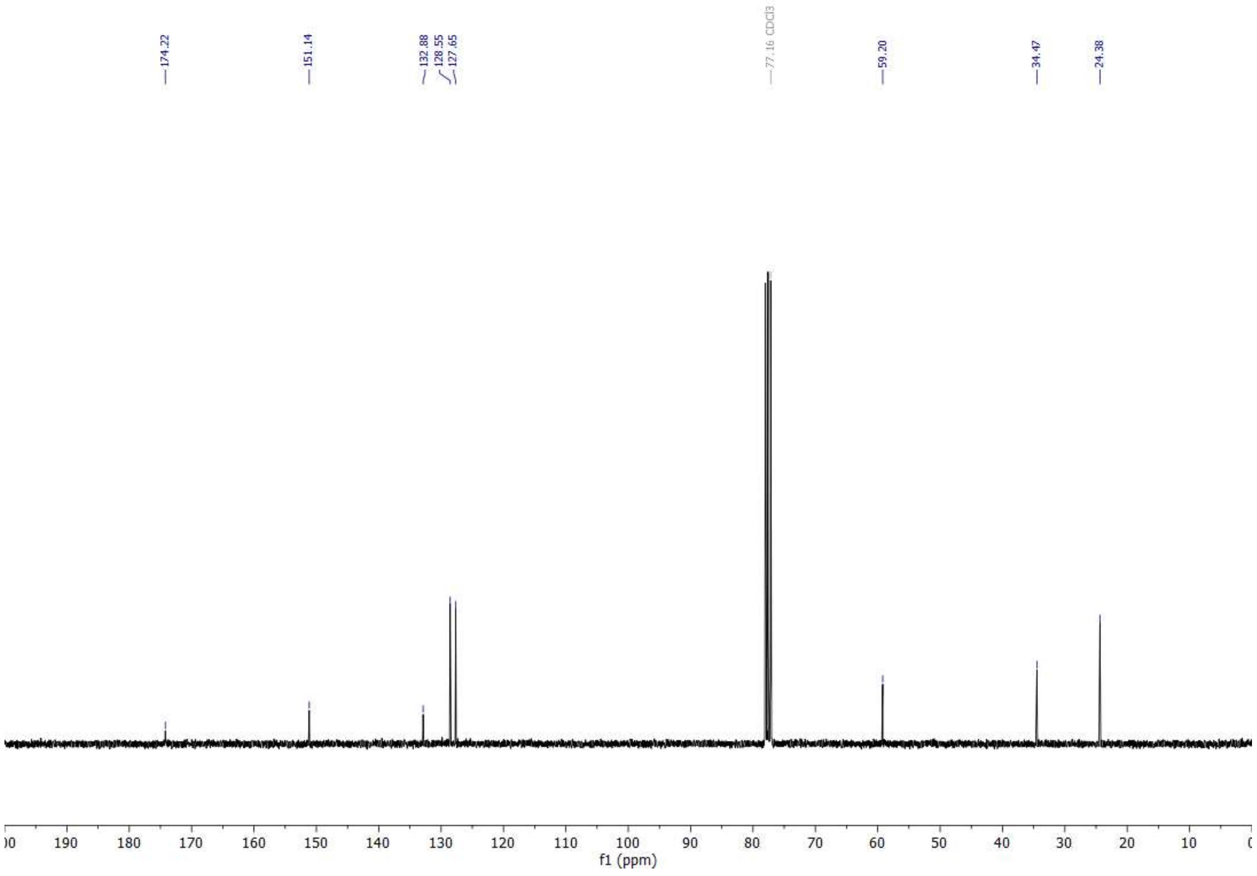
174.22
151.14
132.88
128.55
127.65
77.16 CDCl3
59.20
34.47
24.38
f1 (ppm)

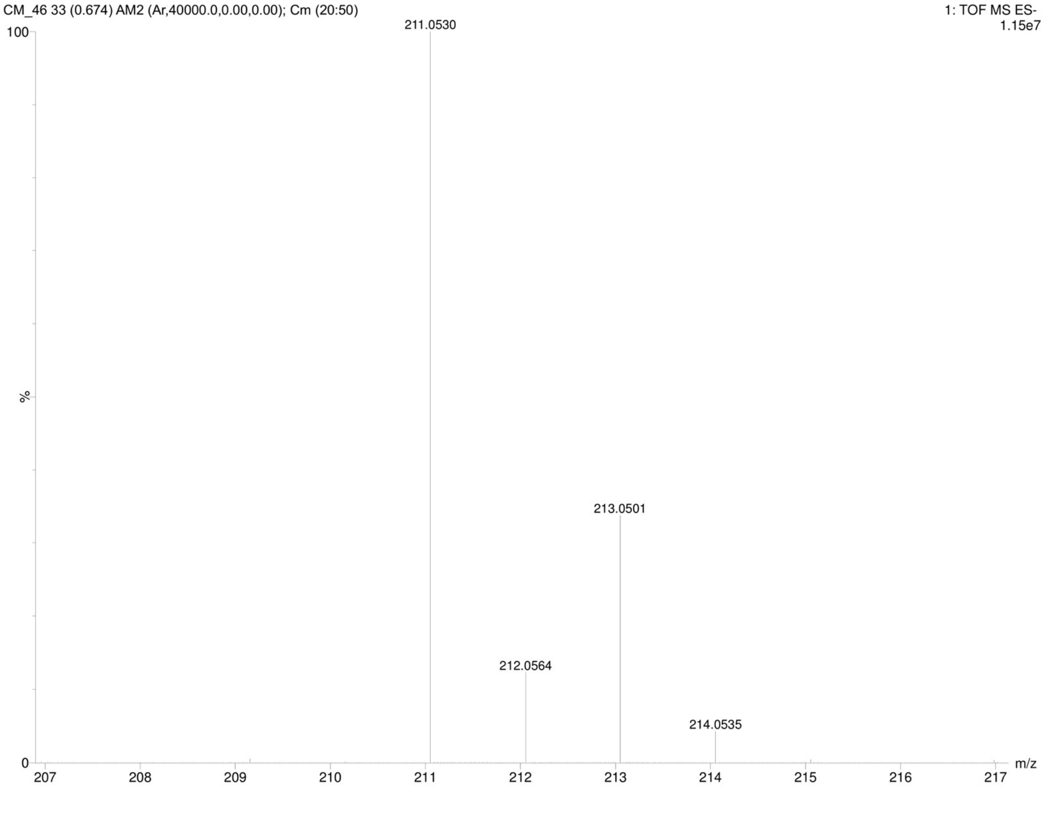
CM_46 33 (0.674) AM2 (Ar,40000.0,0.00,0.00); Cm (20:50)
1: TOF MS ES-
1.15e7
211.0530
213.0501
212.0564
214.0535
%
m/z
