# Supplementary material for: Efficient α-selective chlorination of phenylacetic acid and its para-substituted analogues
Source: RSC Adv. 2025 Apr 17;15(16):12298–303. doi: 10.1039/d5ra00198f (PMC12004119; doi:10.1039/d5ra00198f)
Supplement: RA-015-D5RA00198F-s002 [file RA-015-D5RA00198F-s002.pdf]

Supplementary Information (SI) for RSC Advances.
This journal is © The Royal Society of Chemistry 2025

Compound Characterisation Checklist

ORGANIC CHEMISTRY FRONTIERS

Manuscript ID:

Submitting Author: Cristiano Bolchi

Manuscript Title: Efficient α-Selective Chlorination of Phenylacetic Acid and its para-Substituted Analogues

1. Characterisation of new and known compounds should be reported in line with the data requirements of the journal: https://rsc.li/2Jdig5s
2. For each compound, enter the compound number as per manuscript in the first column, and type a single "**X**" in the cell corresponding to the type of data reported in the manuscript or ESI.
3. Print the completed checklist as a "**.pdf**" file. During submission, upload the saved file together with your manuscript, and select "**Compound Characterization Checklist**" as the file designation.
4. If no synthesis is reported in the paper, please indicate this in the yellow box and upload the form as above

	**I confirm that no synthesis or characterisation of compounds is reported in this manuscript.**

Compound number	New	Known with reference(s) cited	Yield	Physical state/mp if cryst. Solid	IR	UV-Vis	1H NMR	13C NMR	2D NMR	Copy of NMR spectrum in ESI	MS	HRMS	Elemental Analysis	Specific optical rotation	GC/ HPLC	Copy of chromatogram in ESI	X-ray (ORTEP and CIF in ESI)	Notes
1a		X	X	X			X	X		X								
2a	X		X	X			X	X		X		X						
3a		X		X			X			X								isolated as a crude
4a		X	X	X			X	X		X		X						
5a	X		X	X			X	X		X		X						
6a		X		X			X	X		X								
7a		X		X			X	X		X								
8a		X		X			X	X		X								
9a	X			X			X	X		X		X						
10a		X		X			X	X		X								
11a	X			X			X	X		X		X						

Manuscript ID:

Compound number	New	Known with reference(s) cited	Yield	Physical state/mp if cryst. solid	IR	UV-Vis	1H NMR	13C NMR	2D NMR	Copy of NMR spectrum in ESI	MS	HRMS	Elemental Analysis	Specific optical rotation	GC/ HPLC	Copy of chromatogram in ESI	X-ray (ORTEP and CIF in ESI)	Notes
